# Maintaining a Cognitive Map in Darkness: The Need to Fuse Boundary Knowledge with Path Integration

**DOI:** 10.1371/journal.pcbi.1002651

**Published:** 2012-08-16

**Authors:** Allen Cheung, David Ball, Michael Milford, Gordon Wyeth, Janet Wiles

**Affiliations:** 1 The University of Queensland, Queensland Brain Institute, Brisbane, Queensland, Australia; 2 Queensland University of Technology, School of Electrical Engineering and Computer Science, Science and Engineering Faculty, Brisbane, Queensland, Australia; 3 The University of Queensland, School of Information Technology and Electrical Engineering, Brisbane, Queensland, Australia; Indiana University, United States of America

## Abstract

Spatial navigation requires the processing of complex, disparate and often ambiguous sensory data. The neurocomputations underpinning this vital ability remain poorly understood. Controversy remains as to whether multimodal sensory information must be combined into a unified representation, consistent with Tolman's “cognitive map”, or whether differential activation of independent navigation modules suffice to explain observed navigation behaviour. Here we demonstrate that key neural correlates of spatial navigation in darkness cannot be explained if the path integration system acted independently of boundary (landmark) information. *In vivo* recordings demonstrate that the rodent head direction (HD) system becomes unstable within three minutes without vision. In contrast, rodents maintain stable place fields and grid fields for over half an hour without vision. Using a simple HD error model, we show analytically that idiothetic path integration (iPI) alone cannot be used to maintain any stable place representation beyond two to three minutes. We then use a measure of place stability based on information theoretic principles to prove that featureless boundaries alone cannot be used to improve localization above chance level. Having shown that neither iPI nor boundaries alone are sufficient, we then address the question of whether their combination is sufficient and – we conjecture – necessary to maintain place stability for prolonged periods without vision. We addressed this question in simulations and robot experiments using a navigation model comprising of a particle filter and boundary map. The model replicates published experimental results on place field and grid field stability without vision, and makes testable predictions including place field splitting and grid field rescaling if the true arena geometry differs from the acquired boundary map. We discuss our findings in light of current theories of animal navigation and neuronal computation, and elaborate on their implications and significance for the design, analysis and interpretation of experiments.

## Author Summary

Do animals need “cognitive maps“? One of the main difficulties in answering this question is finding a definitive scenario where having and not having a “cognitive map“ result in measurably different outcomes. Many key predictions made by models involving some sort of “cognitive map“ can also be replicated by models without a “cognitive map“. Here we consider published data on rodents navigating in darkness inside homogeneous arenas. The head direction system becomes unstable within three minutes in darkness, yet place and grid cells have been reported to fire in the same locations for thirty minutes or longer. We show firstly that it is theoretically implausible for path integration alone to maintain a stable positional representation beyond three minutes, given a drifting head direction system in darkness. Secondly, we prove that even assuming perfect boundary knowledge is insufficient to maintain a stable positional representation. Finally, we show in simulated and real arenas that a nearoptimal combination of path integration and boundary representation is sufficient to produce stable positional representations in darkness consistent with published data. The necessity for fusing path integration and landmark information for accurate localization in darkness is both consistent with, and motivates the existence of, “cognitive maps.“

## Introduction

### A “Cognitive Map” Is Multimodal but Not Modular

In 1948, Tolman employed two analogies to describe the prevailing classes of models used to explain the experimental data on maze navigation and learning obtained from rats [1]. Tolman likened the stimulus-response class of models to an old fashioned telephone exchange, where incoming calls are linked via connecting switches to outgoing messages. Stimulus-response connections which result in reward are strengthened. In contrast, Tolman was a proponent of the field theoretic or cognitive map class of models, in which the telephone switchboard was replaced by a “map control room”. Tolman asserted that sensory inputs “are usually worked over and elaborated in the central control room into a tentative, cognitive-like map of the environment”. The core issue seems to be whether animals (including humans) acquire and use a unified, multimodal spatial representation for navigation. Alternatively, can a model without a cognitive-like map of the environment explain animal navigation data?

One of the most ubiquitous navigation strategies in the animal kingdom is path integration (PI), a process by which an animal uses an estimate of self-motion to update its location estimate [2]–[4]. PI works in principle under most environmental conditions. There is abundant theoretical and experimental evidence that PI requires stable allothetic directional information in combination with idiothetic motion cues [5]–[11]. Hence in general, PI is likely to be a multimodal process which combines a mix of information from vision, proprioception, vestibular or inertial organs, motor efference copy, and other sources depending on species. Therefore that the PI state is itself a multimodal representation. For example, it was recently shown in humans that PI output depends on a combination of visual and idiothetic motion cues in combination, not independently [12]. Clearly, experimental data is consistent with the multimodality property of a “cognitive map”.

However, it is conceivable that a representation of the world is multimodal and yet modular, and hence fragmented. Recently, an insect-inspired model was proposed in which the navigation system consisted of independent modules [13]. During navigation, each active module produced a directional output, which fed into a recurrent neural network to output an overall heading direction. Behaviours such as shortcutting and landmark-guided homing were successfully explained using this model. Importantly, the authors argued that by maintaining modularity, this model did not require a “map control room” and hence did not resort to a “cognitive map” to explain a number of important navigation behaviours which have previously been used to argue in favor of a “cognitive map” [1], [14]–[17]. Once acquired, a fragmented neural representation of the world seemed sufficient for effective navigation.

The above model highlights the distinction between a “cognitive map” and “map information” i.e., the ability to deduce position of an animal or landmarks from a neuronal ensemble code does not guarantee the existence of a “cognitive map”. It is possible to infer current position from either the PI module or landmark units of [13], and even reconstruct an approximate map of the traversed environment. Hence “map information” was clearly present in the model, despite the absence of a unified “cognitive map”.

An important aspect of the “cognitive map” debate is where an animal's neural system lies along a spectrum spanning complete modularity to full information fusion. In simplistic terms, one may consider neural systems and neural models as being less or more “map-like” according to the degree of information fusion. Focusing on this one aspect of the complex debate, it is clear that even the navigation modules of [13] implicitly assume information fusion from various sensory channels, and may be considered as having some map-like characteristics. On the other hand, there is clear anatomical and functional evidence for information segregation within both vertebrate and invertebrate brains, suggesting that full information fusion is neither necessary nor advantageous.

In this paper, we focus on whether navigation modules as per [13] can, at least in principle, be used for effective navigation inside arenas with featureless boundary walls, in the absence of visual cues. Furthermore, we determine whether or to what extent fusion of iPI and boundary information may improve localization under these challenging conditions.

### The Difficulty of Understanding Visual Navigation

Most behavioural and *in vivo* recording navigation experiments are conducted under light. Information from visual directional cues may lead to superior accuracy and precision in localization and navigation simply by allowing more accurate PI (see later).

Moreover, the advantage of using visual cues is inextricably confounded by the fact that other spatial information is also implicitly present in visual scenes. In both real and simulated arenas, rat-like navigation behaviours may be replicated without explicitly extracting any spatial layout information about the arena, simply by storing and comparing low resolution views [18], [19]. Therefore, the presence of visual information may improve navigation performance in a number of interrelated ways, including PI performance. To circumvent this complication, we focus on a subset of experimental scenarios where visual information is absent or minimized.

### The Difficulty of Path Integration without Vision

PI can provide an animal with a continuous location estimate, even when environmental cues are ambiguous or transiently absent. In practice, PI is subject to the accumulation of errors over time, whose error magnitude has been shown to be critically dependent on the computations used for updating the state of the PI system [11], as well as the directional information which is used [8], [9].

Some classes of PI models have been shown theoretically to be intolerant to noise [11]. In general, two necessary conditions for noise-tolerant PI are an allocentric reference frame (world-centered), and static directional representations. An allocentric Cartesian PI system (e.g. [20]) is one example, where the axes are bound to world-centered directions. Importantly, there are at least two computational subclasses which satisfy both criteria, of which one is “ring-like” and one is “map-like” [11]. Therefore, a “cognitive-map” is sufficient but not necessary for accurate PI in an open field. Conversely, the need for accurate open field PI argues neither for or against the existence of a “cognitive-map”.

In an open field, accurate PI using noisy idiothetic information (termed iPI) is impossible beyond a few steps [8], [9]. In contrast, equally noisy compass information may be combined with idiothetic speed estimation for allothetic path integration (aPI), preserving accuracy, and with significantly smaller positional variance [8], [9]. Hence vestibular, proprioceptive and motor efferent signals are insufficient for open field PI, whereas vision, magnetoreception or other allothetic sensory channels are typically required. Hence even with a “map-like” PI system, the absence of visual or other compass information prevents accurate PI, raising the question of whether iPI can be used as an effective navigation strategy at all.

### Boundaries as Landmarks

PI and landmark navigation are complementary processes. Irrespective of the sensory information or neural circuitry, PI requires calibration by using cues in the environment to correct for errors built up during the PI process. Here, we call any set of cues which vary with location to be “landmarks”. Animals can use a wide variety of landmark cues (e.g. visual, auditory, olfactory, tactile), which provide a mixture of positional and directional information for a given environment. Some landmarks are uniquely associated with a location or orientation in the world while others are less specific. According to [13], the association of a PI state (vector) with each independent unique landmark results in an array of landmark units in memory which serves to guide navigation, without requiring a “cognitive map”.

Boundaries may be considered a subclass of landmarks characterized by their geometric nature, but not associated with one specific point location. There is evidence that neural processing of boundaries may differ from other landmarks [21],[22]. It remains an open question how a navigation algorithm could use boundary landmarks which restricts an animal's path, but which provides no other identifying information [23]. In the present work, we focus on the use of boundary information, not the process of its acquisition.

### Neural Correlates of Navigation Accuracy

Neurons which are preferentially active in particular positions or orientations in space provide a quantitative indicator of the stability of the animal's navigation system. In particular, if a neuron exists whose activity shows spatial selectivity that is stable over time, then it follows that computations required to maintain stable spatial selectivity must occur somewhere in the navigation system.

In the rodent literature, at least four major functional classes of spatially-selective neurons have been identified. Hippocampal place cells [24] encode the rodent's location, cortical and subcortical head direction cells [25], [26] encode the rodent's orientation, medial entorhinal grid cells [27], [28] encode a multiplicity of regularly spaced rodent locations. There are also medial entorhinal border cells [29] and subicular boundary vector cells [30], [31] which both encode the rat's relative location to barriers or boundaries. A subtype of the medial entorhinal grid cells encodes *pose* (conjunctive location and orientation) [28], [32], [33]. In the presence of visual information, the functional relationships between spatially-selective cell types are complex and intimately related to both task and available cues (reviewed by [34]–[37]).

### Head-Direction Tracking Is Unstable without Vision

A number of rat brain regions have been identified containing cells which represent head direction, and which form an interconnected head direction (HD) system [38]. The rate at which the HD tracking system degrades in darkness has been the subject of several studies [39]–[41]. Three important properties have been reported: 1) significant drift occurred after two minutes, 2) the angular deviation distribution was approximately zero-mean and symmetrical, and 3) the absolute angular deviation between consecutive two-minute sessions did not change significantly over time. These three observations suggest that the HD system drifts randomly and approximately at a uniform rate in the absence of vision.

### Place Tracking Is Relatively Stable without Vision

In contrast to the head direction tracking system, place and grid fields remain stable for half an hour or more during active exploration in a dark environment devoid of visual cues. Rat grid fields have been reported to remain stable in round arenas for up to thirty minutes in darkness [28]. Blind rats can generate and maintain stable place fields following exploration of stable landmarks placed within a round arena [42]. However, olfactory and tactile cues were not actively minimized in either study. In a follow up experiment to [42], it was shown that even if odor cues were actively removed by cleaning of the arena floor, 10% of place fields remained stable, and about 50% remained, even over a period of 48 minutes in darkness [43]. Similarly, mice CA1 place fields were found to be stable in darkness in a 1.5 m diameter circular water maze, where floor odour cues were unlikely to be present. Place field stability was observed for two consecutive twelve-minute sessions [44].

Taken together, the above evidence suggest that vision is not essential for the rodent navigation system, for upwards of half an hour. Over short distances, iPI undoubtedly plays a role in navigation without vision [45]. However, given a head direction system which shows appreciable error (drift) beyond the first two minutes in darkness, can iPI explain place or grid field stability in the medium (5–10 minutes [46], [47]) to long term (>30 minutes [28], [42], [43])? Alternatively, can an independent landmark module, perhaps containing boundary information, be used to maintain stable place fields? In fact, can any model assuming only iPI and boundary information explain place and grid field stability in darkness?

In summary, there is an active research field considering how PI interacts with environmental information. However, to date we are not aware of any studies which take a quantitative approach to studying the errors of iPI in arenas, the information provided by the arena geometry, or whether observed neuronal properties can be explained without fusing iPI and boundary information.

### Accurate Localization without Vision

We propose that a stable estimate of location can be maintained by animals for over half an hour without vision, by optimally combining idiothetic motion cues with a featureless boundary map – akin to Tolman's “cognitive map”, but contrary to [13]. We first model the accumulation of errors using only iPI. Using analytical derivations and simulations we show this iPI model cannot maintain place and grid field stability, assuming realistic neural tracking accuracies. Next, we present theoretical arguments showing that using arena boundary geometry without PI is insufficient for localization. Together these results show that any model which uses a modular or decentralized navigation system, including [13], is incompatible with rodent neural data.

Finally, we show using computer simulations and robot experiments how iPI in combination with boundary sensing and a geometric map enables long term stability of a location estimate in a number of arena shape configurations, demonstrating similarity to published experimental results. We demonstrate that the stability of simulated place fields depends on both the arena shape and size. A number of predictions are made regarding the behaviour of place and grid fields under environmental manipulations in darkness, which depend on the way in which iPI and boundary information is used. We discuss these results relating to known neuronal properties, implications on mammalian navigation models, as well as the design and interpretation of experiments.

## Methods

The rationale for the models and simulations in this paper are as follows. Firstly, we calculated the HD error based on the assumption that the HD firing is highly correlated [25], [26], [38] so that the drift in an individual HD cell's tuning function is representative of the error in the HD system. A simple HD model was developed assuming independent Gaussian errors. Rat trajectories were modelled as correlated random walks to traverse each arena homogeneously.

Secondly, we modelled boundary information based on the assumption that each animal had acquired an accurate metric boundary map, prior to removal of visual cues. This ideal assumption was used to estimate the maximum place stability afforded by the boundary.

Thirdly, we tested the plausibility of achieving a stable representation of place by (a) using iPI or (b) the boundary map independently, versus (c) using a near-optimal combination of both using a particle filter approach. We used a range of relevant metrics to compare outcomes from each approach including a dynamic place stability index obtained from the particle filter, simulated place and grid fields, and direct mathematical analysis of positional uncertainty based on principles of PI.

Since noise is inherent in sensory systems, we assumed that when in contact with the boundary, the navigation system had a noisy estimate of the relative angle of incidence to the boundary tangent. Noise was incorporated to mimic measurement imperfections of the biological sensory systems which may be involved in boundary detection. The addition of noise resulted in a small decrease in the place stability of the navigation system (comparison data not shown). We hypothesize that the whisker system may play an important role here, but we have not explicitly modelled a particular sensory system to simulate boundary contact.

For completeness, we also tested a fourth model (d), corresponding to the hypothetical condition that the rat has a perfect memory of the arena boundary, but cannot discern whether it is in contact with the boundary or not. Finally, we tested the particle filter algorithm on a robot platform (e), the iRat, as proof of concept that the navigation model works under real world conditions.

### Simulated Rat and Arenas

Arena sizes and geometries were based on published experiments. In all simulations, circular arenas had a 76 cm inner diameter, corresponding to published experiments [40], [42], [43], [46]. Unless otherwise specified, we used square arenas of the same area (67.4 cm width) for comparison. Other rectangular arenas are individually specified.

Since the arena walls were assumed to be homogeneous, the simulated rat was unable to identify which wall (or wall segment) it was close to. Therefore, wall contact information *per se* did not provide positional information beyond the fact that the simulated animal was somewhere along the boundary.

**Figure 1:**
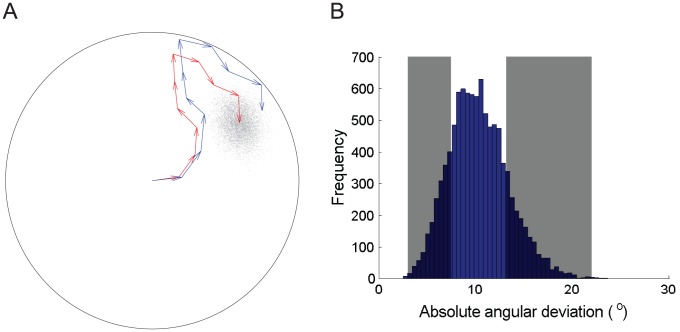
Simulated rat head direction (HD) error in the absence of vision. A. Simulated 10 steps in a 76 cm diameter circular arena, showing ground truth (blue) and pure odometry (red), where cumulative HD error is modelled as a Wiener process, discretized stepwise. The particle cloud estimate of current position (grey) is also shown (see text for details of trajectory model and particle filter). The rate of error variance increase was estimated from [40]. B. Using the same parameters as A, a frequency histogram of absolute angular drift (in °/min) from 10^4^ random paths is shown. From this distribution, 10^4^ samples of size 19 were randomly drawn with replacement, and 95% confidence intervals for the range minimum and maximum are shown in grey. This provides an independent comparison with [41] who reported that a sample of 19 HDCs showed an absolute drift rate ranging from 5.1 to 26.6 °/min without vision. These results suggest that using a discretized Wiener process as a first approximation of rat HD angular drift is reasonable.

Individual rat trajectories were described by a discrete time 2D correlated random walk model, with boundaries (Fig. 1A). Simulated rats walked on average 5.4 m per minute. See Text S1 for trajectory simulation details, and Video S1, Video S2 & Fig. S9 for an example of a simulated 48 minute trajectory.

### Simulated Inputs to the Rat's Path Integration System

The errors in direction and speed estimates for the PI system were modelled as Gaussian random variables. The HD system was assumed to drift coherently but randomly, resulting in the PI system only having access to a single erroneous estimate of head rotation per step. From the results of [40], and the trajectory model described above, the HD error standard deviation was estimated to be approximately 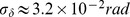 per step or 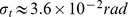 per second (Text S2).

In the absence of direct experimental data, we assumed that linear step size estimation error was normally distributed with 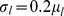 and independent of the angular displacement estimation error. Note that linear displacement estimation error makes a relatively small contribution to the overall positional uncertainty using iPI. For example, assuming straight line navigation in an open field using the error model described, linear errors account for approximately 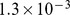
% of the asymptotic rate of positional variance increase (substituting the error model parameters into the results of [8]).

### A Particle Filter Approximation of Ideal Positional Distribution

A particle filter model was used to approximate the Bayes-optimal combination of boundary and iPI information. A rat moving randomly in an enclosed arena will make contact with the boundary sporadically, in principle allowing it to localize to a region close to the boundary. Wall contact can also provide distance and orientation relative to the wall. In brief, the particle filter approximated the pose uncertainty distribution of the simulated rat during iPI through a population of pose estimates (particle cloud). A particle cloud represented a finite sample from the true pose distribution. Each particle may be considered as one possible pose (conjunctive position and heading), and its history may be considered as the simulation of one possible trajectory. During iPI, the stepwise increase in true pose uncertainty was modelled by randomly drawing values from the HD and step size estimation error distributions described earlier, and added to each particle's pose.

Knowledge of the boundary limited the positional spread of the particle distribution, while boundary contact further reduced the unlikely particle pose estimates. Particles were weighted according to the likelihood that their pose explained current sensory (or memory) information, and then the particle population was redistributed according to particle weights. The resulting particle cloud provided a distributed estimate of current pose, having combined arena memory and arena contact information. See Text S3, Fig. S3 and [48] for further details.

The standard stochastic universal resampling procedure was used to update the particle cloud. In principle, this procedure produces a particle distribution which approaches the Bayes-optimal posterior distribution (overviewed in Text S3). Mathematically, this property is only guaranteed if the error models are available and correct. In simulations, these error models were assumed to be available to the rat's navigation system. Empirically, however, small deviations did not appear to cause large differences in the place stability index or simulated place and grid fields. For example, in the iRat experiments (see later), neither the wheel odometric errors nor IR range sensor errors were precisely known. In the particle filter variant used for the iRat, the wheel odometric errors were overestimates, while the IR range sensor error magnitudes were not explicitly used. The particles were effectively ranked based on their relative consistency with sensory data, and a fixed fraction were culled during wall contact (see Text S3 for further details).

In those scenarios where the test arena differed from the training arena, a variant of stochastic resampling was also used for comparison with the standard form. The variant followed the standard method until the final step of assigning pose to the new particle cloud on boundary contact (see Text S3 for further details). In this variant, only new heading was assigned, preserving the particle's original position estimate. When the test and training arenas were identical, this variant was inferior at localization compared to standard universal resampling. However, when the two arenas differed, this particle filter variant avoided large jumps in overall position estimates, and generated tessellating grid-like fields in a greater number of scenarios (Results, Text S12).

### Measuring Place Stability

To provide a performance metric for the particle filter navigation model which accounted for both accuracy and precision, we devised a simple intuitive index of position estimation stability, termed place stability. The mean squared distance of the particles to the true position is affected by the spread of the distribution (precision) and any systematic drift of the particle cloud (accuracy). From information theoretic principles, the baseline is assumed to be a uniform distribution of particles throughout the arena (maximum entropy). The place stability index at each time point is defined as


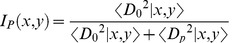
(5)

where 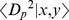 is the expected squared distance of the particles given a true current position 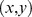, and 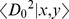 is the expected squared distance between a uniform distribution of particles and 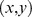. Using squared distances results in simple analytic solutions of 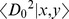 and 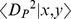 for circular and rectangular arenas (Text S4, Table S1). A performance index of 1 implies a positional distribution equivalent to a Dirac delta function at the true location, while a uniformly distributed hypothesis of position results in an index of 0.5 (chance). Indices below 0.5 may occur if the spread of the distribution exceeds the arena area, or if there is negative spatial correlation. The latter may occur, for example, if the spatial representation is rotated 180° about the center of the arena, relative to the true position.

Since the simulated rat trajectories covered the whole arena homogeneously, it was possible to derive the expected place stability index given boundary contacts (Text S4, Table S2).

### Simulated Place Fields

To understand how the particle cloud representation of place or the place stability index may relate to place fields, a simple model was used to simulate Poisson spike probabilities.

The probability of a spike following each step was modelled as a Binomial process with 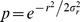 where 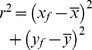. The spike probability decreases monotonically from unity according to the distance *r* between the center of the particle cloud and ideal firing position 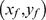. The center of the particle cloud was treated as the center of mass or Cartesian mean, i.e., 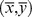. This is also the position which minimizes the squared distance to all particles. In all simulations, 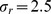 cm, corresponding to the size of the pixel of analysis (e.g. [43]). The size of 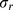 was chosen to be sufficiently large to allow an adequate number of spikes to be generated during a simulated experiment, while being sufficiently small relative to the spatial resolution of the analysis procedure so that 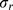 did not dominate the spatial spread of simulated spikes. Although it is somewhat arbitrary what constitutes an adequate spike count for analysis, we aimed to have approximately the same number of spikes as analysis pixels or higher (788 analysis pixels in the circular arena), in the majority of 8 minute periods and field locations studied. This was to avoid spuriously high spatial information values from low spike counts. For instance, one spike per pixel spread randomly across half of the analysis pixels yields a raw Skaggs spatial information content of approximately 1 bit/spike. The latter results from a low spike count rather than true spatial specificity.

In addition to using the Cartesian mean, place field simulations were repeated using the polar mean for the circular arena simulations (Fig. S5). The polar coordinates of the particles were first averaged to give 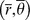. Here, the angular mean 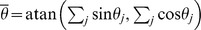 where 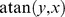 denotes the 4-quadrant arctangent. The Cartesian coordinates of 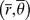 was used as a substitute for 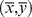. The polar mean was used due to the fact that the particle cloud distribution in circular arenas tended to follow a crescent shape approximately aligned with the circular boundary (discussed further in Text S9, see Video S1 & S2 for an example). Under these conditions, the polar mean was a good approximation of the modal position of the distribution. The Cartesian mean was often close to or even within the concavity of the crescent-shaped distribution, rather than near the mode. This caused an underestimation of the radial position of the cloud. However, the cloud distribution tended to be a convex shape in rectangular arenas, so there was little difference between the two methods near rectangular boundaries. The polar mean was not used throughout the simulations because the estimation of radial distance close to the arena center was contaminated by the spread of the cloud (Text S9).

Over a period of time, the simulated spike pattern represents a temporal average reflecting a sequence of complex particle cloud states. These states in turn depended nonlinearly on the actual trajectory taken, the boundary information gained, and random errors. Therefore, place stability changed dynamically during each trial, and affected the spatial specificity of the simulated spike sequence depending on location 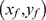.

Both positional and angular specificity were quantified using Skaggs information [49] to be comparable to published data on place fields. A maximum likelihood factorial model [50] was applied to check whether decoupling of positional and directional information affected the estimated spatial information content.

### Simulated Grid Fields

Since our results showed that the particle filter output had a complex dependence on the pose distribution and wall information, we investigated the effect of using an arena boundary representation different to the one being traversed. This is analogous to a change in arena size and/or aspect ratio while in darkness.

With vision, rat grid fields have been reported to rescale when rats are transferred between rectangular arenas of different size and aspect ratios [51].

Grid fields were modelled as multiple independent place fields distributed as a regular hexagonal tessellating array over the entire training arena. Grid fields were simulated by assuming that the firing probability was determined by 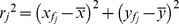 where 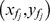 was the position of mode *j* of the grid field. Similar to place fields, the firing probability of each contributing subfield was given by 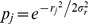. Following each step, the maximum allowable number of spikes was capped at one. It was assumed the training arena's boundary representation remained in memory during all tests.

### The iRat – Localization in a Real Arena without Vision

**Figure 2:**
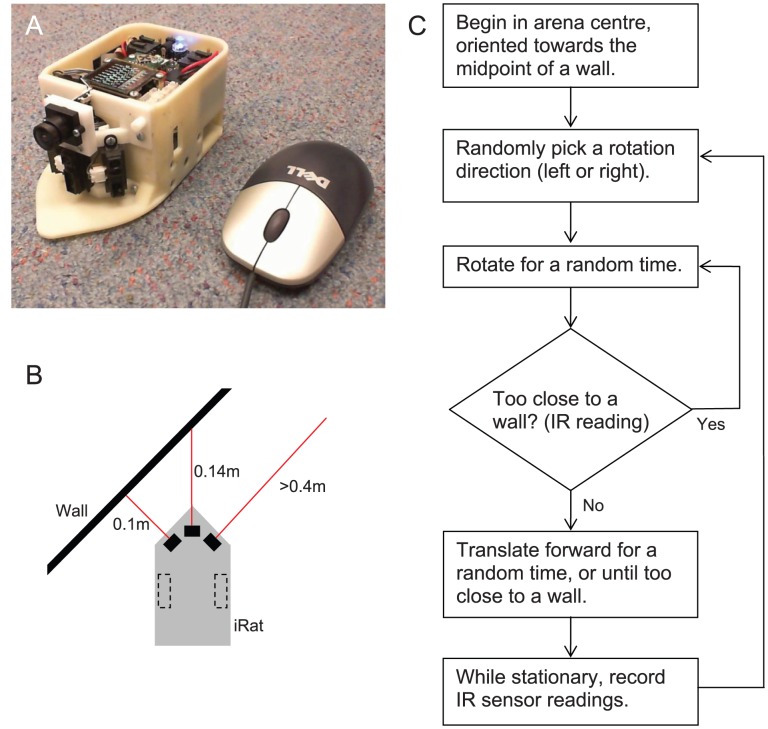
The Intelligent Rat Animat Technology (iRat). A. Prototype iRat (left) shown next to a standard computer mouse (right). The Sharp IR sensors are oriented at −45, 0, +45 degrees relative to the midline (black rectangular components near the base of the robot, below the midline camera). Image obtained from [52]. B. Schematic illustration of IR sensor locations and readings. C. Flow diagram for controlling the iRat's trajectory.

To show that the derived and computer simulation results can be applied in real environments, we used the prototype iRat [52], [53], Intelligent Rat Animat Technology robot for experiments in real arenas (Fig. 2A). The iRat is comparable in size and mass to a laboratory rat at 150 mm×80 mm×70 mm at 0.56 kg. The iRat has a camera, speakers and microphone, on board computation via 1 GHz PC, WLAN, and IR (infrared) proximity sensors. The IR sensors may be considered as providing crude ‘whisker’ information near walls. In this study, only the three IR sensors were used to obtain three distance estimates when close to arena walls (Fig. 2B), whereas the camera was not used. See Text S5 for details of the iRat experiments, and Text S3 for details on the particle filter variant.

## Results

In the following sections, we present results and analyses which examined the feasibility of using independent iPI and landmark modules [13] for localization without vision. We first characterized the performance of iPI using the HD error model developed from empirical data as described in Methods. Then we quantified the estimation error in using only a featureless boundary for localization, to determine whether a boundary landmark module suffices to maintain stable navigation in darkness. Next we combined iPI with boundary information using the particle filter approach described in Methods, to determine whether or by how much the estimate of position improved. Finally, a series of unimodal and polymodal firing fields were simulated using a particle filter to mimic place fields and grid fields under various experimental conditions. These simulations tested whether it is computationally plausible for observed place and grid field stability to be maintained for 30 minutes or more using only iPI and a featureless boundary map, given an erroneous HD system.

### Limits of Idiothetic Path Integration in an Open Field

Using the simplest description of locomotion which consists of a turn and step, it has been shown previously that the asymptotic rate of increase in positional variance per step is 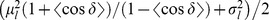 where 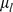 is the mean step length, 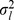 is the variance of the step length, and 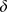 is the angular error per step [8]. This result was derived assuming iPI along a straight course in an open field. For a zero-mean, normally distributed 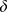, 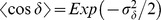 where 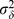 is the variance of the HD angular error per step. For ease of interpretation, the variance rates in this section are reported in terms of time rather than steps.

**Figure 3:**
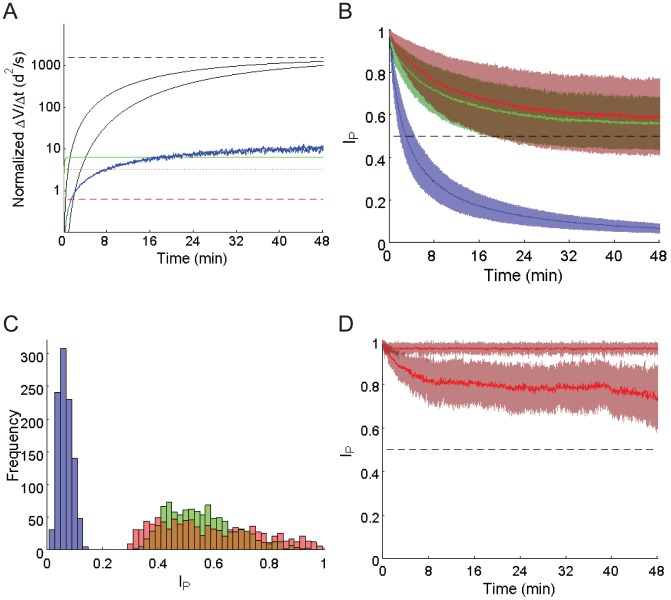
Place stability without vision in a circular arena. A. Predicted rate of positional variance change (ΔV/Δt) using iPI in an open field with small HD error (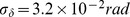, black solid lines – consistent with rat HD tuning error without vision) and moderate HD error (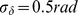, green solid lines – approximately equal to intended turns of the simulated trajectories inside experimental arenas). Asymptotic variance rates (dashed lines) are shown. From simulation (blue lines), the positional variance rates (with respect to true position) were found assuming an intended trajectory with moderate tortuosity (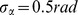), and heading error 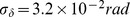. Limits defining navigation failure (dotted and dashed red lines) are shown. Intentional and erroneous turn distributions are Gaussian, with constant step size 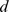. See text for further details. B. Place stability index values (mean ± s.d., 10^3^ trials) in a circular arena of 76 cm radius. The conditions were iPI only (blue), iPI and boundary memory (green), iPI and boundary memory and contact information (red). Chance level is shown (dashed line). See Methods for details on the simulation of quasi-random trajectories. C. Frequency histogram of place stability values following 48 minutes without vision (colour code as per B). D. Comparison of the top 10% of place stability in square (upper line) and circular (lower line) arenas, with boundary memory and boundary contact information (mean ± s.d., 10^2^ trials).

Let 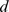 be the mean distance travelled per second. Since 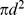 is the area of the traversable region within one second, iPI errors are considered irrecoverable if the positional variance increased beyond this rate. This is because the true position may be anywhere within an area too large to be traversed even in theory. Note that 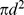 represents a highly optimistic threshold since one unit of positional variance encompass less than half of all possible positions in a circular bivariate Gaussian distribution (Fig. 3A red dotted line). For a 95% confidence region, the threshold is 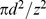 where 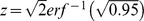 (Fig. 3A red dashed line). Even with this correction, the threshold is optimistic since it represents the limit of search recovery (assuming error-free search) and could not plausibly sustain a stable place representation. Nevertheless, it allows estimation of a loose upper bound on the time limit of the use of iPI.

Combining the results of [8] with the HD model in our current simulations, we determined whether it is theoretically plausible for iPI to maintain an accurate long term estimate of position. Assuming the HD cell error model described in Methods, without any step length estimation error (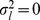), the predicted asymptotic positional variance increased at 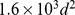 per second (Fig. 3A black dashed line). Since 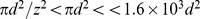, the positional uncertainty increased much faster than the maximum area which can be traversed, clearly showing that iPI cannot be used to accurately track movement along a straight trajectory in the long term.

In the short to medium term, positional variances of iPI increases more slowly than the asymptotic rate [8], [9]. Substituting the HD error model parameters into the exact variance expressions derived in [8], the optimistic limit of 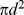 was exceeded after 51 seconds (perpendicular to axis of intended locomotion), and 192 seconds (along the axis of intended locomotion), again demonstrating that iPI became irrecoverably inaccurate well within 1 to 3 minutes from the start (Fig. 3A black solid lines).

### Limits of Idiothetic Path Integration in an Arena

Next we considered tortuous trajectories where the intended path had directional variance 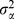 (Methods and Text S1). Clearly, a rat trained to forage within a small arena has to change direction regularly, following a tortuous rather than straight course. This occurs, for example, in many experiments within confined arenas.

Path tortuosity may decrease the rate of positional variance increase in two ways. Firstly, if an iPI system is unable to track the actual turns, then the HD error angle would be dominated by the physical turn angle i.e., 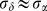. For the path tortuosity used in the simulations in this work, the predicted asymptotic positional variance increase was 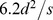 (Fig. 3A green dashed line), still greater than the conservative limit of 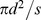. The limit of 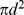 was exceeded within 10 seconds from the start of iPI (Fig. 3A green solid lines).

Another possibility is that path structure itself influences the way in which a small cumulative HD error 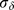 impacts on the position estimate. This was modelled as an unbounded correlated random walk [6] with path directional standard deviation 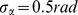 and HD error 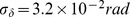. Since closed form solutions to these variance functions are not available (but see [6] for empirical approximations), Monte Carlo simulations were performed. The positional variance rate remained within the limit of 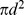 for nearly eight minutes (Fig. 3A blue lines). However, the 95% confidence interval exceeded the limit of 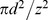 in 88 seconds, making accurate iPI impossible within the first one and a half minutes. Nevertheless, an iPI system with small HD error can track a tortuous path more accurately than a straight path, showing that path structure itself can affect navigation performance.

Inside an arena, animal paths are further constrained by the boundary. Since boundaries force animals to make turns that they would otherwise not need to in an open field, we expected that positional variance would increase more slowly inside bounded arenas, for a given baseline tortuosity of the intended trajectory. We tested this prediction using the trajectory model described in Methods and Text S1. The maximum average rate of position variance increase was found to be less than 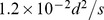. At first, this may seem to support iPI as a plausible process to maintain place stability inside bounded arenas. On more careful analysis, this was found not to be the case.

Assuming a Gaussian distribution, we found that the 95% confidence area of the positional error distribution exceeded the entire area of the arena by 175 seconds. To quantitatively track the accuracy and precision of the position estimate of individual trajectories, we used the place stability index 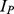 (Methods, Text S4). In a circular arena, the average 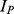 over 10^3^ simulations fell below chance (0.5) within 179 seconds (Fig. 3B). Similar results were obtained for the square arena (184 seconds - see Fig. S6B). This means that despite a much reduced rate of increase of positional variance, the best estimate of position afforded by iPI is no better than chance level beyond 3 minutes. In other words, the navigation system has no useful information about its true location within the arena beyond 3 minutes without vision.

Taking a third approach, we quantified the maximum spatial information of the position estimate itself, assuming a Gaussian field centered in a circular arena, and the trajectory and HD models described earlier. It was found that the corresponding spatial information fell below 1 bit after 150 seconds using iPI alone (Text S6, Fig. S1). The maximum spatial information content of any firing field will be expected to fall below 1 bit per spike in under 3 minutes without vision. In comparison, some place fields in the “dark+cleaning” condition of [42] showed spatial information of about 1 bit/spike or higher over three consecutive 16 minute windows.

In summary, iPI alone cannot sustain a useful position estimate beyond 2 to 3 minutes without vision in either open field or enclosed arenas. Intended path tortuosity and arenas can both reduce the magnitude of positional uncertainty, with implications for the comparison of results obtained in confined arenas and open fields.

Interestingly, even aPI was unable to maintain place stability or generate a stable place field if used alone (Text S11, Fig. S7, Table S4). Assuming the same HD error distribution as above, but reset following each step to mimic having stable distant visual landmarks, the time taken for the average place stability index to drop below chance level (0.5) was increased but still unable to explain stable place or grid fields beyond approximately 5 minutes.

### Boundary Proximity Alone Is Uninformative

The rat's navigation system was assumed to have continual access to information about whether it was in contact with the arena boundary or not. We investigated whether this information alone can increase the place stability index above chance level, assuming error-free knowledge of the arena size and shape.

When wall contact has occurred, denoted by *W+*, the ideal posterior positional distribution is a narrow region along the perimeter of the arena, since positions closer to the center of the arena should not result in wall contact. We investigated the possibility that this information alone may have increased place stability above chance by finding 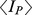 when wall contact has occurred. The range and expected *I_P_* values can be found assuming a uniform sampling of the perimeter, and assuming an idealized uniform posterior distribution following wall contact, corresponding to the perimeter line (see Text S4 and S7 for further details).

Under the above assumptions, in any square arena, 
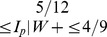
 while the expected or average 
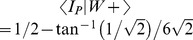
. For any circular arena, 
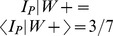
. Note that these indices are independent of arena size. When not in contact with the boundary, denoted *W−*, the animal may be anywhere within the arena giving 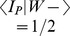.

Thus, in the absence of PI information, the expected place stability index does not exceed chance level (0.5) even assuming ideal information about the arena boundary (summarized in Table S2). Hence above-chance place stability cannot be achieved only using arena boundary information.

An alternative interpretation of the above results is as follows. Suppose that a randomly foraging rat occasionally contacts the arena boundary. Mostly, the rat knows it is not at the boundary, so its internal representation is a uniform distribution over the entire arena, called the null position estimate. On boundary contact, its internal representation of position becomes a uniform distribution along the featureless boundary, called the boundary position estimate.

We ask whether the boundary position estimate improves the estimate of current position when the rat is actually at the boundary. It can be shown that for all convex arena boundaries, the estimation error is actually greater if the animal used the boundary estimate compared to the null estimate (see Text S7 & Fig. S2 for proof). This conclusion applies to all functions of position estimation error which increase monotonically with the estimation error distance. As an example, the mean squared position estimation error of an animal at the boundary of a circular arena using the boundary estimate is 33% larger than the null estimate (Table S1). Therefore, using the boundary geometry actually increases the mean squared position estimation error compared with using the null estimate.

### Localization Estimates Are Stable in Darkness Using Boundary Memory and Wall Contact Information

Inside arenas, positional uncertainty may be modified by the use of boundary information. Two types of information are considered: a) boundary memory only; and b) boundary memory plus wall contact information. The latter may be due to whiskers or other haptic information and was assumed to provide approximate wall distance and incident angle (Methods). In both the circular (Fig. 3B–D) and square arena (Fig. 3D, S6B & S6D), the average place stability index remained above chance for 48 minutes when arena boundary information was used. On average, using wall contact information improved place stability. In contrast, place stability dropped below chance within the first 8 minute window, in the absence of arena boundary information (Fig. 3B, S6B blue lines). Boundary information in the square arena consistently improved average place stability beyond that in circular arenas. This pattern of results persisted when only the most stable 10% of trials in the two arenas were considered (Fig. 3D).

The average place stability indices remained above chance level for 48 minutes without vision in both circular and square arenas. Since it was shown earlier that neither iPI nor boundary information alone could achieve this, the current particle filter implementation demonstrates greater place stability than independent iPI and boundary landmark modules.

For completeness, we consider the possibility that an animal's navigation system can switch between functional modularity and information fusion. We therefore ask whether an occasional switch to using a modular navigation system as per [13] may still produce a similar level of place stability, provided that optimal information fusion occurs at all other times. It can be shown that following a single boundary contact using a modular navigation system, the maximum expected place stability 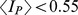 (proof in Text S8). Although this value is marginally higher than chance (0.5), it is significantly lower than near-optimal fusion of iPI and boundary information after 48 minutes without vision (t_999_ = 11.17, p<10^−100^). Most importantly, the uncertainty distribution becomes a circular annulus concentric with the boundary, incompatible with any stable place field restricted to one sector of the arena. This mechanism can, however, maintain a stable place field at the center of the arena. Overall, even occasional use of a modular navigation system causes a significant decline in place stability, incompatible with stable place fields (other than at the arena center).

### Simulated Place Fields Are Stable in Darkness Using Boundary Memory and Wall Contact Information

**Figure 4:**
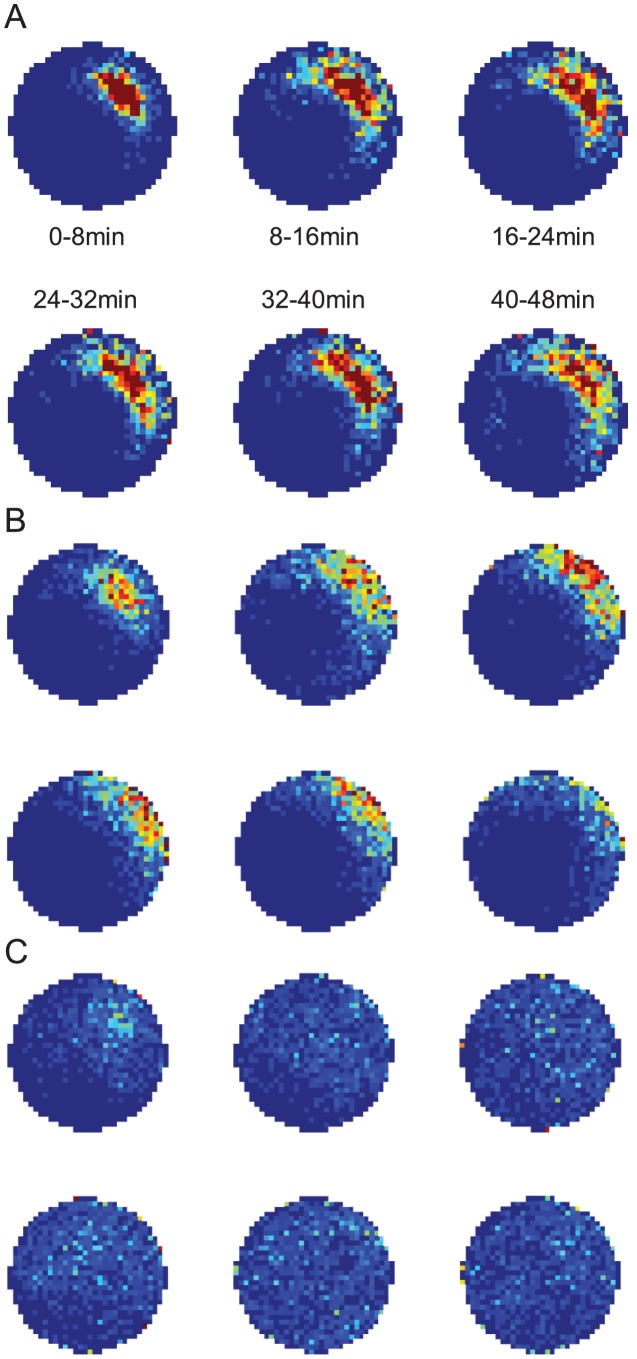
Simulated place fields without vision in circular arenas. The average of the most stable 10% of simulated place fields are shown using arena geometry, boundary contact and iPI information (A), using arena geometry and iPI information only (B), and using iPI only (C). All colour scales are set at a maximum value of 0.15 spikes/step.

Despite the higher place stability index with fused iPI and boundary information, the average place stability decreased continually over 48 minutes without vision in the circular arena. This decrease was clear even when the most stable 10% of trials were considered (Fig. 3D). We investigated whether the decreasing place stability could support stable firing fields, using a Poisson probability model (see Methods for details). Fig. 4A shows the pooled average firing field generated during consecutive 8 minute time windows, for the 10% of trials with the highest place stability indices (to be comparable to the results of [43]). A more extensive set of simulated place field locations are shown in Fig. S4 & S5 corresponding to using the Cartesian and polar mean, respectively, to estimate particle cloud position (see Methods and Text S9 for details).

During each 8-minute time window, the firing fields were quantified using five metrics: 1) the spatial information content; 2) the directional information content; 3) the number of spikes; 4) the spatial correlation coefficient calculated bin by bin, relative to the first 8-minute window; and 5) the spatial coherence [54] (Table 1). The information content was calculated using the maximum likelihood factorial model [50]. The bin sizes used were 2.5 cm by 2.5 cm for position and 6° for direction.

**Table 1:** Simulated place field properties.

iPI, boundary memory and wall contact information						
Property	Time (minutes)					
	0–8	8–16	16–24	24–32	32–40	40–48
*Spatial information	2.8	2.1	2.1	2.2	2.2	1.8
*Directional information	0.053	0.052	0.062	0.044	0.039	0.055
No. Spikes	804	932	942	929	952	965
†Spatial correlation	1	0.74	0.71	0.72	0.63	0.52
Coherence	0.94	0.90	0.88	0.88	0.86	0.85

iPI and boundary memory but no wall contact information						
Property	Time (minutes)					
	0–8	8–16	16–24	24–32	32–40	40–48
*Spatial information	1.7	1.4	1.4	0.92	1.0	0.88
*Directional information	0.079	0.055	0.086	0.062	0.054	0.077
No. Spikes	506	588	531	686	715	700
†Spatial correlation	1	0.42	0.32	0.088	0.16	−0.046
Coherence	0.67	0.56	0.45	0.11	0.31	0.12

* Information units in bits/spike.

† Bin-wise Pearson correlation coefficient.

With boundary memory and wall contact information, the spatial information content remained close to 2 bits per spike for 48 minutes without vision, while the directional information content remained at less than 0.1 bit per spike. There was also high field coherence and the spatial correlation remained above 0.5. Using the polar estimate of position, the spatial correlation remained above 0.8 for all fields and time periods except those at 30 cm (Table S3). The high spatial correlation is comparable to rat place fields in circular arenas of the same diameter, in the presence of visual information (R = 0.70 [55]). Together, the simulation results show that in the circular arena, stable place fields are maintained for 48 minutes without vision, in at least 10% of trials, similar to experiment [43].

Place fields were considered stable by [43] as those which rotated by less than 12° between the control period and the first test period. This was estimated by rotating fields from each time window, about the arena center, to find the maximum spatial correlation R_max_ possible. This angular displacement, Δθ_max_, is indicative of one possible way in which place fields may become unstable. Using the same analysis method but at 1° rather than 6° resolution, and using 8-minute instead of 16-minute time windows, we found that the most stable 10% of place fields rotated by 12° or less between the first period of no vision, compared with each of the subsequent periods, compatible with experiment (see also Table S3). These results further support the current model as a reasonable approximation of the computations carried out by the rat navigation system.

For completeness, we tested whether boundary contact *per se* was beneficial (Fig. 3B, Fig. 4B, Table 1). The same procedures were used, but without boundary contact information. In the absence of boundary contact information, the spatial information content was substantially and consistently lower than with boundary contact (see Fig. S6 and Text S10 for square arenas). Similarly, the spatial correlation was below that of having boundary contact information, for all time windows. In particular, the spatial correlation for the 8–16 minutes without boundary contact information was below that of the 40–48 minute time window with boundary contact information showing a significant, immediate and persistent decline in place field correlation compared to the first 8-minute period. The spatial firing pattern was less stable without arena boundary contact information.

Hence boundary memory was useful in culling particles which went outside the boundary extent, thereby limiting the growth of the uncertainty distribution (see also Text S3). This contrasts from pure iPI, where the particle cloud width increased without limit. However, particles within the arena but far from the boundary were never culled in the absence of boundary contact information, even if their pose estimates were otherwise highly inconsistent with the sensory information from boundary contact. Thus boundary contact *per se* may be considered as providing information which, when used appropriately, can reduce positional uncertainty beyond that of having arena memory only.

Consistent with analyses presented earlier, simulations using iPI alone resulted in no stable place fields (Fig. 4C). Due to extremely low spike counts even with pooling across trials, the spatial information content could not be estimated reliably.

### Simulated Place Fields with Changing Arenas

A common experimental manipulation for testing neural and behavioural properties in navigation tasks is to change the arena size and shape. In simulation, this was achieved by explicitly specifying a different arena size and/or shape to that which was traversed. In this way, the arena in memory may be considered as that acquired during training, while the test arena is introduced at the beginning of each trial, at the moment when visual information becomes unavailable.

**Figure 5:**
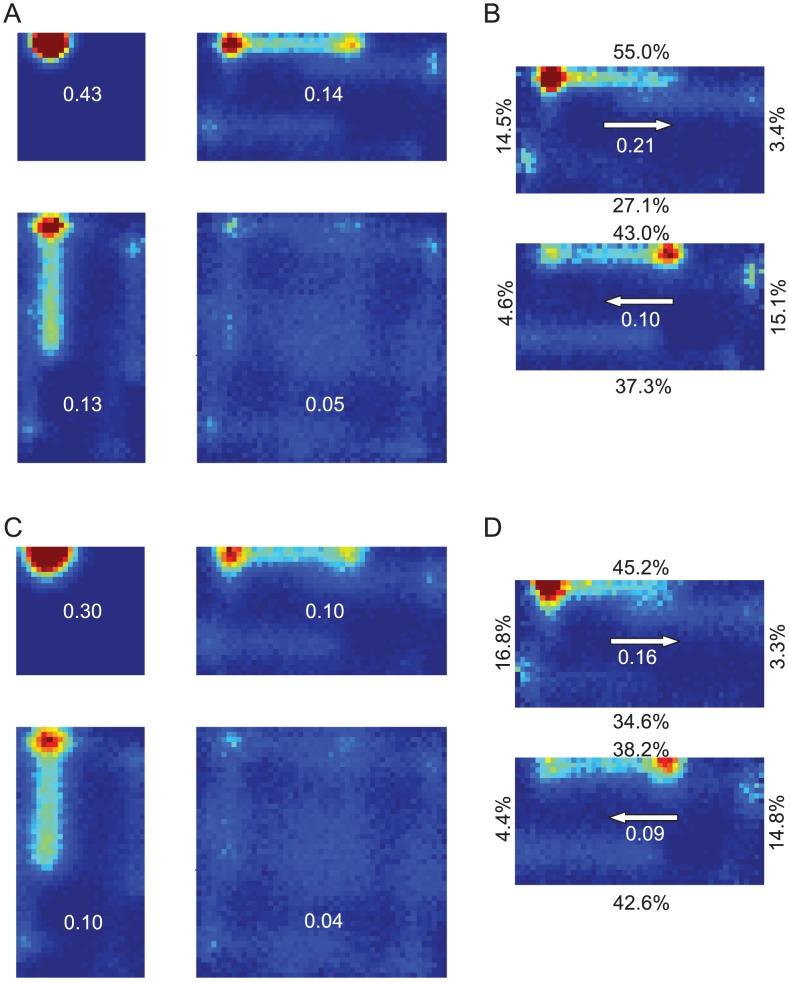
Simulated place fields without vision in changing rectangular arenas. Simulated place fields were generated using standard stochastic universal resampling of particle pose (A) or stochastic resampling of heading only (C), pooled from 10^3^ random trials over 48 minutes without vision (see Methods). Training was assumed to occur in a square arena 61 cm in width, while testing occurred in 61 cm by 61 cm (A & C, upper left panels), 61 cm by 122 cm (A & C, upper right panels), 122 cm by 61 cm (A & C, lower left panels), and 122 cm by 122 cm (A & C, lower right panels) arenas. The fields in the upper right panels of A & C (61 cm by 122 cm test arena) are decomposed based on heading (B & D respectively). The average firing fields are shown when the simulated rat's heading had an allocentric easterly (B & D, upper panels) or westerly (B & D, lower panels) component (assuming upwards in each diagram is North). The percentages indicate the relative frequency of each wall being the most recently contacted prior to each spike in the field. The maximum firing rate is indicated for each field (spikes/step).

It has been shown that some place fields established in a square arena either stretched or split when the arena geometry was changed [56]. More detailed analysis of the split fields showed that the firing subfields had different modal positions depending on the direction of travel of the rat. Although these experimental results were obtained with vision, we tested the effect of the same arena geometry manipulations without vision as predicted by the two particle filter models described in Methods (Fig. 5).

Using the same place field model as described in Methods, we tested the effect of having a different traversable arena to that stored in memory. Directional information content was low in all cases (Table 2).

**Table 2:** Information content with changing arenas.

Resampling	Test Arena Dimensions (cm)	61 by 61	61 by 122	122 by 61	122 by 122
*Standard	Spatial information	4.1	1.1	1.1	0.26
	Directional information	0.0060	0.0092	0.0065	0.083
*Heading only	Spatial information	3.3	0.80	1.0	0.24
	Directional information	0.15	0.020	0.084	0.0062

* Pooled from 1,000 random trials.

Place field stretching or splitting was found in the three novel test arenas, with the emergence of directional selectivity in the split fields similar to [56]. In our simulations, the spatial information content decreased by more than 2 bits/spike between the training arena (61 cm by 61 cm, Fig. 5A & 5C upper left panels) and horizontal rectangular arena (61 cm by 122 cm, Fig. 5A & 5C upper right panels), without a concomitant change in directional information. Hence the directional selectivity of the individual modes of the bimodal firing field (Fig. 5B & 5D) cannot be attributed to a change in the overall directional selectivity of the field.

To determine whether the most recent wall contact may be related to the pattern of firing, the frequency of each immediately preceding wall contact was found (Fig. 5B & 5D). In both fields, the highest frequency of recent contact was of the top wall, which was also the nearest wall. The two particle filter variants used yielded similar results. The largest relative differences in frequencies were of recent contacts with the left and right walls with over threefold changes consistently. In particular, during rightward traversals (Fig. 5B & 5D upper panels) spikes were preceded most recently by left wall contacts more frequently than right, while during leftward traversals, (Fig. 5B & 5D lower panels) spikes were preceded most recently by right wall contacts. These results show that the proximity of boundaries is correlated with the relative frequency of most recent contact, determined retrospectively from each spike.

The marked differences in the relative frequencies when fields are divided based on rightward versus leftward trajectories can be explained as follows. Due to the temporal correlation of headings along simulated trajectories, a leftward trajectory is more likely to have recently come from the right part of the arena, and vice versa. Therefore, a leftward trajectory was more likely to be preceded most recently by contact with the right wall than the left wall, and vice versa. In the results shown in Fig. 5, the arena representation in memory was a 61 cm by 61 cm square, and the place field was positioned 15.5 cm from the left wall and 45.5 cm from the right wall. Therefore during testing in the 61 cm by 122 cm arena, rightward trajectories resulted in maximal firing approximately 15.5 cm from the left wall (Fig. 5B & 5D upper panels), while leftward trajectories resulted in maximal firing approximately 45.5 cm from the right wall (Fig. 5B & 5D lower panels).

When the test arena was a 122 cm by 122 cm square (Fig. 5A & 5B lower right panels), the split fields showed particularly low spatial information content (<0.3 bits/spike) using either particle filter variant. The low spatial specificity was due to the large discrepancy between the dimensions of the training and test arenas in both spatial dimensions (see Text S12 & Fig. S8 for further details on error mechanisms).

### Simulated Grid Fields with Changing Arenas

With vision, grid field spacing has previously been shown to partially rescale along the direction of a rectangular arena which is stretched or compressed varying with the arena geometry transformation [43]. The rescaling factor in grid spacing was consistently less than that of the arena rescaling. We tested the same arena transformations using the particle filter model variants described in Methods.

**Figure 6:**
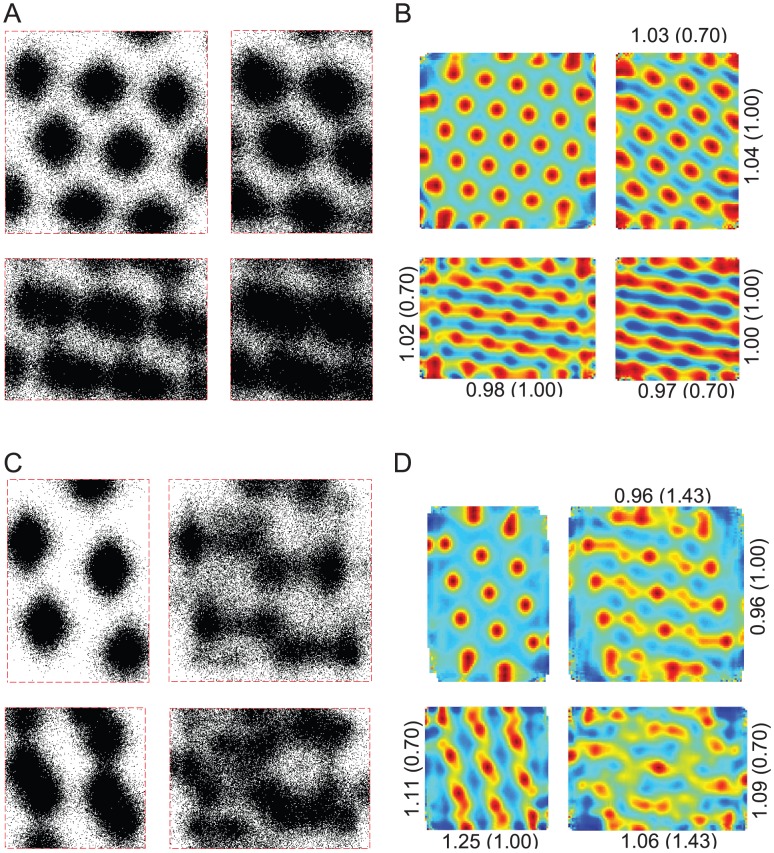
Simulated grid fields without vision using standard stochastic universal resampling. Simulated grid fields were generated using standard stochastic universal resampling of particle pose (see Methods), pooled from 10^3^ random trials over 48 minutes without vision. (A) Training was assumed to have occurred in a 100 cm by 100 cm arena. Testing in a 100 cm by 100 cm arena (top left), a 100 cm by 70 cm arena (top right), a 70 cm by 100 cm arena (lower left), and a 70 cm by 70 cm arena (lower right). Points indicate mock firing locations from 1,000 trials of 48 min in darkness. (B) Spatial autocorrelograms of grid fields in (A). Rescaling indices are shown for each vertical and horizontal axis, with the true environmental rescaling factor shown in parentheses. (C) Training was assumed to have occurred in a 100 cm by 70 cm arena. Testing in a 100 cm by 70 cm arena (top left), a 100 cm by 100 cm arena (top right), a 70 cm by 70 cm arena (lower left), and a 70 cm by 100 cm arena (lower right). Otherwise as per (A). (D) Spatial autocorrelograms of grid fields in (C).

**Figure 7:**
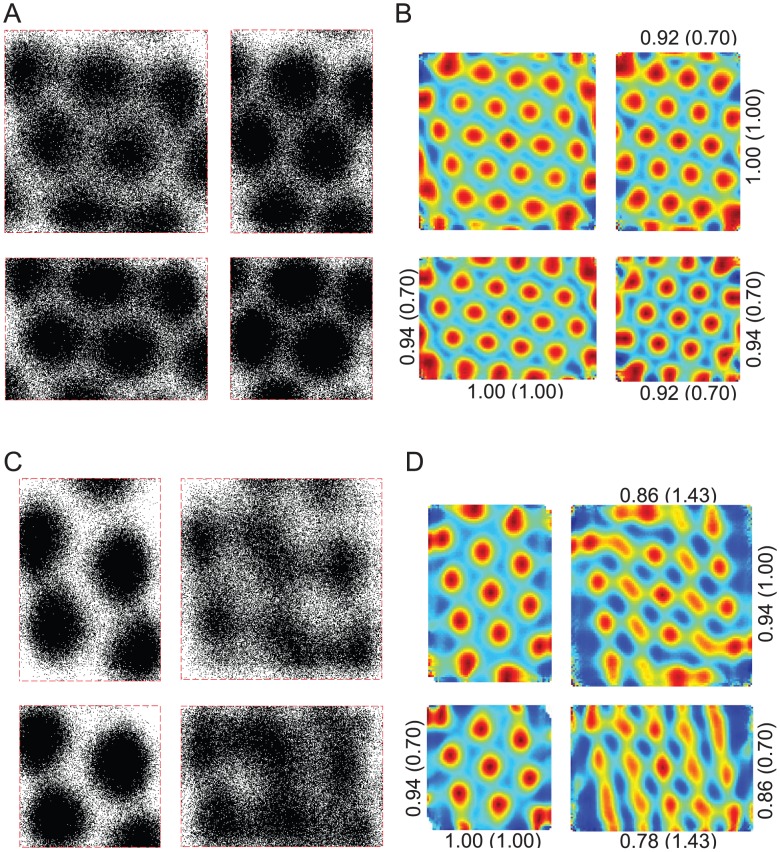
Simulated grid fields without vision using stochastic resampling of particle heading. Using a variant of the standard particle filter (see Methods), only particle heading was resampled. All other details are as per Fig. 6.

Firstly, we found that an unstable HD system (e.g. without vision) can maintain a variety of stable grid-like firing fields, even if the test arena differed to the training arena in geometry (Fig. 6 & 7). Secondly, using particle resetting of heading only, arena compression caused a partial rescaling of grid spacing (Fig. 7A & 7B), in a manner qualitatively similar to that observed *in vivo*, with vision. The magnitude of the partial rescaling was less than reported (approximately 25% of the arena rescaling, compared to 48% reported by [51]).

Grid rescaling did not occur in simulations where arenas were stretched (Fig. 6C, 6D, 7C, 7D). Instead, grid field splitting was seen - analogous to the phenomenon of place field splitting reported earlier.

It must be emphasized that the primary purpose of simulating arena manipulations was to test whether it is possible for stable place and grid fields to be maintained without vision, despite different dimensions between the training and test arena. A secondary goal of these simulations was to demonstrate that specific hypotheses about the combination of iPI and boundary information can be modelled using the particle filter approach. The differences in results between the two variants of the particle filter used highlights the importance of determining the precise manner in which information is used for spatial navigation.

### The iRat - A Real World Demonstration

**Figure 8:**
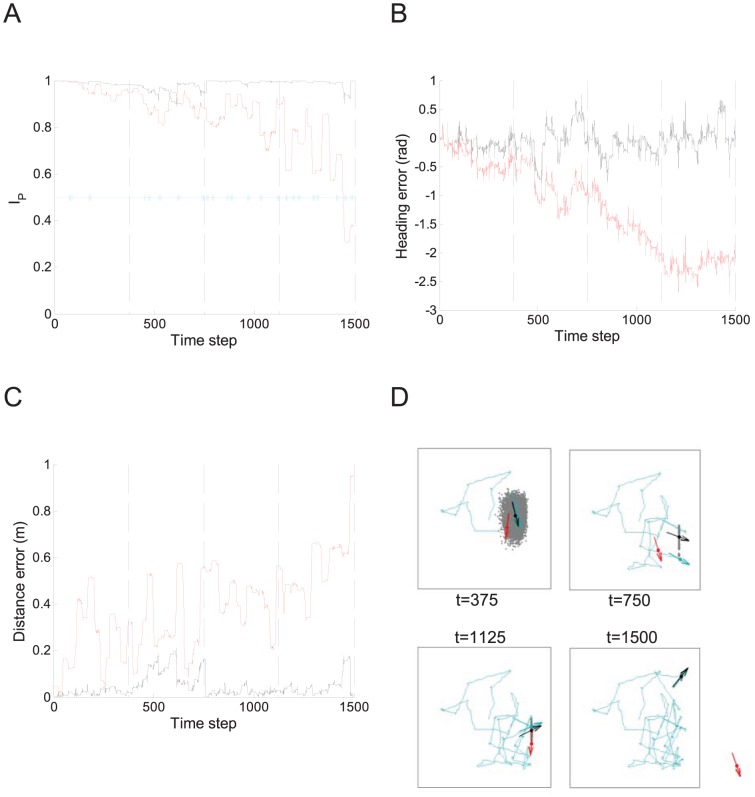
The iRat maintains place stability without vision. (A) Performance of the iRat in a 1 m square arena using a particle filter approximation of the positional uncertainty distribution (black line), compared with pure odometry (red line). Periods where at least one laser whisker registers a distance below 30 cm are shown (cyan bars). The total trajectory time was approximately 5 minutes (1,500 time steps). (B) The error between estimated heading and actual heading. (C) The error between the estimated position and actual position. (D) The ground truth trajectory (blue) is shown following every 375 time steps during a 1,500 time step iRat trial (dashed lines in A to C). The estimated position and orientation from pure odometry (red) is consistently inferior to using arena information (black) via a particle filter (gray dots). See Methods for details of the iRat.

To demonstrate near-optimal navigation without vision in real world conditions, we adapted the particle filter method to a mobile robot platform, the iRat, moving in a real arena (Fig. 8). Cumulative odometric errors caused a gradual drift in the estimate of heading and position, in an analogous way to simulated rodent iPI described earlier, making localization for any prolonged period of time using pure iPI impossible. In contrast, application of the particle filter to combine arena geometry and IR ‘whisker’ information maintained highly accurate localization for the duration of the experiment (5 minutes). All iRat experiments shown were conducted without using the on-board camera.

There was no appreciable decrease in place stability, or increase in pose error, over the trial period. Successful localization using the iRat in a real arena demonstrates that the proposed algorithm is robust and can function without precise knowledge of real world characteristics including iPI estimation errors or wall contact estimation errors (discussed in Text S3 & S5). Egomotion and sensory error characteristics are often difficult to ascertain explicitly by a navigating agent, and may depend on interactions with the environment during a particular navigation trajectory which cannot be known ahead of time. This work also demonstrates the feasibility of using the iRat to test computational algorithms in similar arenas to rodent experiments.

## Discussion

We have used a particle filter approach to combine iPI and boundary information, enabling a probabilistic estimate of position, prior knowledge of the arena boundary geometry, plus relative orientation and distance of wall contact to be fused in a near-optimal way. This approach increased the average place stability index to well above chance for at least 48 minutes without vision, in either a square or circular arena. Furthermore, we have shown that in principle, such a unified multimodal navigation system allows spatially stable firing patterns to be produced despite using a drifting HD system, analogous to stable place and grid fields in darkness. We showed that these results could not be achieved using either iPI or boundary information alone, which means that information fusion must occur. This result is incompatible with functional modularity such as that proposed by [13], while being consistent with the existence of some sort of “cognitive map” in the rodent brain. Related issues and implications are discussed below.

### Is the Head Direction System Really Unstable?

It is possible that differences in experimental conditions account for some of the discrepancy between HD instability and place/grid field stability. The clearest test of this possibility should involve simultaneous recordings of HD cells, place cells and grid cells without vision, but has not been reported to date.

It is possible that odor or other non-visual cues may provide orientation information. Ideally, experiments should be performed with stringent control and quantification of environmental cue signals. But like simultaneous *in vivo* recordings in multiple regions, such experiments may be challenging in practice. It is worth noting that the HD error rate was estimated from experimental data obtained without the active removal of odor cues [40]. Given that place field stability is adversely affected by odor cue removal [43], it is possible that HD system stability may also be affected, potentially making the HD error rate larger than in our model.

On the other hand, tuning of individual cells in the HD system may only be partially correlated, especially across multiple brain regions. If the observed HD error per cell has a random component which varies independently between HD cells, it is possible that the directional information error from the entire HD ensemble may be smaller than predicted based on individual HD cells. Based on published experimental evidence [26], [56], this seems unlikely to be of significance.

If stable place and/or grid fields without vision imply the presence of a near-optimal distributed pose estimate, and this pose estimate is available to the HD network, then in principle, feedback may correct HD cell tuning errors. This seems unlikely in practice. Firstly, there does not seem to be any anatomical or functional evidence reported of feedback correction to the HD cells from either place cells or grid cells. Secondly, our simulations predict that the optimal heading error is small and relatively stable in square arenas (Fig. S6F, S6H), leading to the prediction that a fully corrected HD system should not drift in square arenas without vision. Since the HD system apparently drifts even in a radial arm maze [29], this prediction seems unlikely but remains to be tested. Thirdly, in open field PI with vision or other compass cues, the absolute HD (even if with noise) is more accurate than angular displacement estimates (e.g., refs [8], [9]). This is especially relevant if familiar landmarks are sparse. Hence there is a role for absolute HD, as well as AHV information in a spatial navigation system. An effective head direction system should contain both. Finally, it has been demonstrated previously that conjunctive grid cells have the computational properties needed to represent pose [33]. Since conjunctive grid cells are found in mEC [28], [32], it seems plausible for the pose estimate to be maintained in mEC, and sustain place field stability downstream. It is worth noting that during PI, the correct update of pose is in the direction of translation which may not necessarily be the same as head direction. However, neurons which encode translation direction *per se* have not been reported.

It is also unclear how grid and/or place cells may switch between the types of direction information to use. Although an important question, it is outside the scope of our current modelling efforts. We speculate that removal of visual input may trigger a switch in, or at least modulate, the type of directional information used.

### Olfactory Cues Are Neither Necessary Nor Sufficient for Stable Place Fields

It seems plausible that some olfactory information was present during the place field recordings in the “dark+cleaning” experiments reported by [43]. Despite the arena being cleaned prior to switching off room lights, it was possible that the rats laid down odor cues strategically or accidentally during the 48 minutes in darkness. However, HD cell tuning curves continue to drift under similar conditions [40], [41] suggesting that natural odor cues do not provide absolute orientation information. Furthermore, blind rats do not express stable place fields in circular arenas until at least one haptic landmark is introduced within the arena [42] suggesting that natural odor cues are insufficient to generate stable place fields. More recently, place fields in mice were found to remain stable in darkness in a Morris water maze [44], suggesting that stable ground odor cues are not necessary to maintain stable place fields in darkness. Nonetheless, once formed, the maintenance of stable place fields may be aided by the presence of olfactory cues in the absence of vision [43]. Similarly, olfactory or other allocentric cues may have contributed to the stability of grid fields in darkness reported by [28]. Therefore it is important to note that our work did not assume odor cues to model stable place and grid fields in darkness.

### Using an Unstable Head Direction System to Maintain a Stable Representation of Place

If the head direction system is truly unstable, the HD tuning direction *per se* cannot be used to maintain a stable place representation via PI. This is because angular displacement errors accumulate, which is incompatible with medium to long term localization (Figs. 3, 4, 1, S6, S7; see also [8], [9]). Angular displacement or angular velocity, however, can be used since a probabilistic estimate of pose can be updated without using absolute direction. From moment to moment, this type of navigation may still be considered as an iPI process but with important and frequent corrections using a combination of boundary memory and boundary contact information. The latter provide a mix of conjunctive position and direction information which can be used to improve the distributed estimate of pose in an approximately Bayes-optimal way. It is an approximation because a particle filter, by its nature, is necessarily a discrete approximation of pose even if all filter properties are optimized for known sensory error characteristics.

Computationally, angular displacement or angular velocity estimates may be obtained via differences between consecutive HD readings, or more directly via signals of angular velocity as found in angular head velocity (AHV) cells [38], [57], [58]. In this work, we have assumed that the rate of drift of the entire HD system, including AHV cells, is indicated by the drift in HD tuning functions. It may be the case that AHV signals have a different error rate compared to the difference between HD tuning signals. However, in the absence of vision and other compass cues, it seems highly likely that the HD signal itself is maintained indirectly through an estimate of angular velocity or acceleration.

By providing angular displacement, an erroneous HD system is computationally compatible with a stable place or grid representation. As shown, stable place and grid fields can be simulated using a distributed conjunctive representation of position and orientation (pose), when combined with a coherent representation of the arena boundary. The former is consistent with recent neurorobotic research suggesting that a distributed conjunctive pose representation is required for managing perceptual ambiguity using visual sensing [33]. Here, we have shown that managing perceptual ambiguity without vision can also be achieved using a conjunctive pose representation.

### The Role of a Boundary Map

With vision, it is difficult to disentangle the localizing properties of views from knowledge about the arena boundary geometry. For example, it has been shown previously that complex navigation performance patterns within confined arenas may be explained using view-based gradient descent strategies [18], [19]. These strategies did not require any topological or metric representation of the environment i.e. no “cognitive map”. However, the evidence presented here suggests there may be circumstances where a map-like representation of the local environment is highly advantageous. For example, in darkness and where odor and other allocentric information may be sparse or ambiguous, sporadic contact with natural or artificial boundaries may suffice to maintain place stability for extended periods, until a localizing cue or landmark is detected [42].

In robot navigation research, it has long been known that relatively featureless boundary features such as walls in an office environment can, provided a probabilistic map representation is used, enable a robot equipped with a range sensor to navigate and maintain a correct and stable estimate of its location within the environment [48]. Here, we have applied that principle to situations where boundary information is only available when in close proximity to the boundary, and where local boundary contour does not uniquely identify location.

Recently, [44] reported that the cerebellum is important for PI in mice. This conclusion was based on the finding that impaired cerebellar function impaired place field stability in featureless circular arenas. However, we showed that place stability under such conditions is likely to require a boundary map interacting with the PI system. Therefore, the observed reduction in place field stability in cerebellar mutants could be equally explained computationally by a number of different effects involving this map-PI interaction, not just impaired PI. In particular, mutants showed stable place fields in darkness in the presence of a single haptic boundary landmark – suggesting that iPI was not completely impaired. Our results highlight the importance of using quantitative models to determine the computational demands of specific tasks such as PI. Further experiments will be required to carefully dissect the contribution of arena boundary information during spatial navigation tasks, given the significant role it can play in maintaining a stable representation of position.

An important question which remains to be addressed in future work is how a rodent's navigation system builds a useful representation of the environment. Some biological aspects of this important question has been addressed using blind rats [42]. Without any local landmarks within the circular arena, place fields were not observed. These rats were free to make contact with the boundary wall, but had no memory information, at least initially. Using our existing particle filter model, it is currently impossible to update the iPI estimate of position in the absence of arena memory. Under a Bayesian formulation, this is equivalent to the case where the likelihood term is not available, so the posterior distribution is unchanged. Consequently, the result is similar to pure iPI – where no place field is seen. During exploration, a noisy estimate of pose can in principle be used to build a representation of the boundary. This is the problem of SLAM (simultaneous localization and mapping) which has been studied extensively in robotics. One avenue of future investigation may be to combine rodent-inspired SLAM models (e.g., [33]) with a boundary representation to study questions related to the acquisition of a novel boundary map, including optimal movement strategies.

### Place Stability versus Stable Firing Fields

Using a simple probabilistic place field model, it was found that emergent firing fields were related to a true ensemble representation of place in complex and unexpected ways. Firstly, the location of a place field in an arena affected the rate of decrease of spatial information in the absence of vision. In these simulations, it was found that fields near the center of circular arenas persisted for longer, preserving a higher amount of spatial information than fields closer to the boundary. Furthermore, the average spike rate varied considerably and may even increase for some time as place destabilization proceeded. Secondly, the arena shape *per se*, independent of traversable area, influenced place stability without vision, thereby affecting spatial information content of place fields.

These results have a number of implications. Firstly, Skaggs information should be interpreted carefully in the context of positional stability. It is undoubtedly a useful quantifier of positional or directional specificity, but may not accurately reflect the true accuracy and precision of the underlying navigation system. We have shown that it is theoretically possible for identical trajectories and spatial representations to give rise to different values of spatial information content, depending on place field location.

Secondly, spatial specificity measures like Skaggs information do not distinguish between unimodal and multimodal fields, or spurious results due to extremely low spike counts. The former is confounded by true field splitting [56], while our simulations suggest the peak spike rate itself may be affected by an interaction between field position and arena shape. Conversely, it is clear that a navigation system with high place stability is computationally able to generate a firing field with stable spatial information content. Less intuitively, high spike rates did not always correlate well with high place stability or high spatial information content. For example, using iPI and arena memory only, the spatial information content for each 8 minute window was approximately 1 bit/spike or higher, while the spike count increased by nearly 40% over the 48 minute period (Table 1). The latter result could have been interpreted as indicating a stable representation of place – but this was not the case. Similarly, the spatial information content between 32 to 40 minutes without vision was greater for the simulated place field at 10 cm from the arena center, than between 8 to 16 minutes for the simulated place field at 20 cm from the arena center (Table S3). If these fields were recorded from separate experiments, the result may have been interpreted as indicating differences in the availability and/or use of spatial cues between the experiments, but this was not the case in the simulations.

Thirdly, even though we purposefully modelled the situation where visual information was absent, it is likely that animals use non-visual information when visual information is present, leading to redundancy. It is therefore plausible that neural computational demands may vary substantially from one experimental design to another, depending on the degree of information redundancy. Even controlling for total area, we showed that the shape of an arena affected the navigation performance of the same navigation model (e.g., Fig. 3D, Fig. S6, Table 1 vs Table 2). Furthermore, we have shown there are large differences between iPI in open and enclosed spaces (e.g., Fig. 3A). Consequently, if there is any spatial navigation element to a task in question, either explicitly or implicitly, the available spatial information must be considered carefully, and quantified where possible.

Finally, it is important to note that our models did not make explicit predictions about theta phase [e.g., 59]. There is a systematic relationship between a rat's position within a place or grid field and the theta phase of cell spikes. This raises the possibility that the navigation system has more precise positional information than suggested by the spatial specificity of the entire field. Therefore, the fact that our model predicts spatial information content of over 4 bits/spike in some cases may be due in part to the fact that we have not embedded the positional information in a phase code, which may reduce the apparent spatial information measured in the conventional way. The latter implies that spatial information may need to be considered as a joint function of spike rate and theta phase.

### Implications for Models of Mammalian Path Integration

Current models of mammalian PI [34]–[37], [59] usually assume stable allocentric direction information as input. In contrast, our results show that absolute HD information is not sufficient in the presence of drift, whereas angular displacement information is sufficient for effective navigation in darkness. It should be noted that these results do not prove that angular velocity information *per se* is used, since angular displacement could be inferred from the change in successive absolute HD estimates. However, past experiments have shown that temporary inactivation of the vestibular system disrupts both the location-specific firing of place cells and the direction-specific firing of HD cells despite visual and odor cues being available [60], [61]. Together with the existence of large populations of AHV cells (more populous that HD cells in some regions [38]), these evidence strongly suggest that angular velocity or acceleration information is critical for optimal function in the rodent spatial navigation system. One avenue of future research is to incorporate angular head velocity models calibrated using vision [57], [58] into existing models of mammalian PI.

Specific effects of noise on the performance of the oscillatory interference and attractor classes of mammalian PI models have been considered previously [62], [63]. However, it is clear that allothetic information such as boundary geometry must be used in conjunction with PI for accurate localization, rather than relying on PI in isolation. It will be necessary to investigate the cellular and computational basis for acquiring and using a boundary map. One theoretical approach is to extend the existing mammalian PI models to incorporate a boundary map, and determine whether it is possible to achieve stable place/grid fields assuming realistic sensory inputs and error magnitudes. A second approach is to consider the movement strategies and possible cues which a rat may use to acquire a boundary representation in the first place. Two candidate cell types under modeling investigation are the boundary vector cell [30], [31], [56], [64], [65] and border cell [29]. In conjunction with a position code, boundary neurons may be able to encode an arena boundary shape.

### Need for a Cognitive Map?

The current work focused specifically on situations where allothetic cues were purposefully minimized or removed. Hence it was by design that only boundary cues could reasonably have been expected to provide stable allocentric information. But as [23] stated, “when boundaries are not available, other types of landmarks can be effectively recruited by the mapping system” consistent with [17]. Indeed, our results are restricted to one particular set of scenarios where a “cognitive map” may be the only plausible explanation of biological data, namely place and grid field stability without vision or olfaction. Our systems-level model does not preclude other stable cues from being incorporated if or when they are available (e.g., [33], [66]). Indeed, there are scenarios where a modular navigation system may perform as well as any “cognitive map model” (discussed further in Text S14). Nevertheless, in at least some conditions, our results demonstrate clearly that as separate modules (e.g., [13]), iPI and boundary information are much inferior to a unified, near-optimal combination of both.

Maintaining separation of PI and landmark (in this case a featureless boundary) modules as per [13] cannot support stable place or grid fields without vision or olfaction beyond 2 to 3 minutes. Therefore, to explain observed place and grid cell firing properties, it is *necessary* that PI and landmark information are combined, consistent with Tolman's analogy that inputs are “worked over and elaborated” [1]. We also demonstrated that a near-optimal probabilistic combination of iPI and boundary information is computationally *sufficient* to generate stable place and grid fields without vision or olfaction. If our interpretations of published experimental data are reasonable, it would be difficult for any model without a cognitive-like map to produce stable place or grid fields without vision. The arguments of [13] that insects do not possess a “cognitive map” cannot be extended to rodents, possibly reflecting general differences in the spatial navigation systems of different animal phyla.

An important question is whether the necessity of fusion of iPI and boundary information should be considered as sufficient evidence of a “cognitive map” in rodents. Although this issue is partly one of semantics and definition, we note a number of important points. Firstly, the necessity of information fusion is at odds with the stimulus-response class of models which Tolman used to contrast against the “cognitive map” class of models. Secondly, the fusion of information is consistent with Tolman's notion of a “central control room” in describing a “cognitive map”, where information from various sources is combined in a coherent manner to produce useful output. Thirdly, the fundamental output of PI systems is metric spatial information. If boundary information has to interact in a useful manner with PI information, it must also contain metric information. It is difficult to envisage a useful representation of an arena boundary with embedded metric spatial information, which bears no resemblance to a spatial map. Fourthly, neither fusion of iPI and boundary memory information only, nor intermittent use of a modular navigation system can maintain stable places fields, showing that the use of a “cognitive map” *per se* is not always sufficient under the conditions described. The nature and degree of information fusion in using a “cognitive map” are important. Finally, it has been demonstrated that it is possible for a “cognitive map” model which combines iPI and boundary information in a near-optimal manner to explain stable place and grid fields without vision or olfaction.

### Conclusions

The rodent HD system becomes unstable in darkness beyond 2–3 minutes, consistent with the theory that allocentric cues are required to maintain long term stability. Place cells and grid cells in rats can show stable firing fields for over 30 minutes in darkness. We have shown that these results are incompatible with PI or a boundary representation if they are used independently. However, a “cognitive map” model which combines both can support stable place and grid fields in various arenas while using a drifting HD system. This model predicts place and grid field splitting, and under some conditions grid field rescaling, without vision. The results support the utility of a conjunctive pose representation for optimal navigation, and show how rats might be able to navigate effectively in environments where visual and olfactory cues are unreliable or absent. Seemingly featureless boundaries are powerful landmarks, when combined with a PI mechanism, for stable medium to long term navigation. The influence of such powerful landmarks on navigation tasks must not be underestimated in experimental design or data interpretation.

## Supporting Information

Figure S1 Spatial information content expected from iPI alone. Mean ± s.e.m. of KL divergence of 1,000 trials using HD error model described in Methods, assuming precise linear displacement estimates. See text S5 for further details.
(PDF)

Figure S2 Comparing distances to boundary and interior points of convex shapes. A. Geometric construct showing an arbitrary convex 2D shape (light grey), with a small region (dark grey) indicating all points between point P and segment S along the perimeter. B. Taking the limit as the length of S approaches zero, the dark grey region in (A) is approximated by a scalene triangle. C. An expanded view of the scalene triangle of (B), showing a circular arc of radius w_0_ (dashed line) centred at P.
(PDF)

Figure S3 Place stability estimates using different particle cloud sizes. A. The mean place stability index (*I_p_*) is shown for 100 random trials, in a circular arena of 78 cm diameter, using iPI, arena memory and boundary contact information. The particle filter was updated using stochastic universal resampling, with particle cloud populations ranging from 10^6^ to 10^3^. B. One standard deviation of *I_p_* is shown for the last step of the results in A. Notched boxplots are shown for the root-mean-square (RMS) distance error from the true position, using the Cartesian (C) and polar (D) estimates of position, averaged over 48 minutes in darkness for the same trials as A and B.
(PDF)

Figure S4 Place stability in darkness using the Cartesian mean of the distributed position estimate. The average of the most stable 10% of place fields (n = 100) are shown in 8 min time windows (A, C, E and G). The ideal locations are 0 cm (A), 10 cm (B), 20 cm (C), and 30 cm (D) from the centre of the arena, along a line at 45° from the horizontal. The corresponding heading distributions during spikes (blue) and over the entire period (red) are also shown (B, D, F and H). For the averaged place fields in each of the 4 locations, there was no significant deviation from the assumption of uniform heading distribution at 0.05 level (Rayleigh's test with Bonferroni correction), and the directional information content was an order of magnitude lower than the spatial information content, typically around or below 0.1 bits/spike (Table S3), consistent with the majority of these fields being pure place representations. All pseudocolour scales used a maximum value of 0.15 spikes/step. The results of G & H are discussed further in Text S9.
(PDF)

Figure S5 Place stability in darkness using the polar mean of the distributed position estimate. The average of the most stable 10% of place fields (n = 100) are shown in 8 min time windows (A, C, E and G). The ideal locations are 0 cm (A), 10 cm (B), 20 cm (C), and 30 cm (D) from the centre of the arena, along a line at 45° from the horizontal. The corresponding heading distributions during spikes (blue) and over the entire period (red) are also shown (B, D, F and H). For the averaged place fields in each of the 4 locations, there was no significant deviation from the assumption of uniform heading distribution at 0.05 level (Rayleigh's test with Bonferroni correction), and the directional information content was an order of magnitude lower than the spatial information content, typically around or below 0.1 bits/spike (Table S3), consistent with the majority of these fields being pure place representations. All pseudocolour scales used a maximum value of 0.15 spikes/step. The results of G & H are discussed further in Text S9.
(PDF)

Figure S6 A comparison of localization performance in circular and square arenas without vision. The mean ± s.d. of the place stability index over 48 minutes with vision are shown for using iPI only (blue), iPI and arena memory (green), iPI and arena memory and wall contact information (red) in a circular (A) and square (B) arena. The square arena was the same area as the circular arena which was 76 cm in diameter. The corresponding colour-coded frequency histograms of place stability values at the end of 48 minutes are shown in C (circular arena) and D (square arena). The angular error mean ± 1s.d. and mean ± 2s.d. simulated HD (grey) and particle filter estimate of heading (red) are shown for the circular (E) and square (F) arena, for the top 10% of trials based on place stability index. The angular error mean ± 1s.d. and mean ± 2s.d. simulated HD (grey) and particle filter estimate of heading (red) are shown for the circular (G) and square (H) arena, for a random 10% of trials. For the top 10% trials based on place stability index used to generate place fields in Table S3, the relative frequency distributions of the Cartesian (red) and polar (blue) mean estimates of radial position are shown for the circular (I) and square (J) arena. Each raw count was normalized by dividing by the actual frequency of the simulated rat being in each radial position bin of 1 cm width. The dotted line shows the ideal relative frequency distribution assuming error-free position tracking. The inset shows the relative frequency with respect to X position in the square arena.
(PDF)

Figure S7 Place fields using allothetic path integration (aPI) only. A. Place fields were generated using aPI only. Fields were centred at (0,0) to maximize spatial information content (see S3). Stable place representations could not be maintained using aPI alone. B. Allothetic PI (grey) led to higher average place stability than iPI (blue). Mean ± s.d. of 1,000 trials are shown for each PI model. In a typical case, the place stability index dropped below chance (dashed line) within 8 minutes using aPI alone (on average in under 5 minutes).
(PDF)

Figure S8 The effect of arena transforms on the estimated position in darkness. The mean particle cloud X position is plotted against the true X position from 10 random trials in each of two reciprocal arena transform simulations, using each of two stochastic resampling methods. The training arena was 100 cm by 100 cm (A & B), and 100 cm by 70 cm (C & D). Examples using both the standard stochastic universal resampling method (A & C) and resampling of heading only (B & D) are included for comparison. The colour-coded original (black) and transformed (red) arenas are shown schematically above each corresponding plot.
(PDF)

Figure S9 Place stability index of the example described in Text S12. The corresponding particle cloud dynamics are shown in Video S1 and Video S2 (periods indicated in grey).
(PDF)

Table S1 Mean squared distance errors in circular and square arenas, assuming location at the boundary.
(PDF)

Table S2 Mean place stability index in circular and square arenas.
(PDF)

Table S3 Properties of simulated place fields in a circular arena.
(PDF)

Table S4 Properties of simulated place field using allothetic path integration only.
(PDF)

Text S1 Simulated random trajectories.
(PDF)

Text S2 Head direction error model.
(PDF)

Text S3 Particle filter navigation model.
(PDF)

Text S4 Place stability index.
(PDF)

Text S5 Robot iRat experiment.
(PDF)

Text S6 Estimating the expected spatial information using iPI only.
(PDF)

Text S7 Mean squared distance and general distance measures to convex boundaries.
(PDF)

Text S8 Asymptotic place stability index in a circular arena.
(PDF)

Text S9 Varying simulated place field locations.
(PDF)

Text S10 Maintaining place stability in square versus circular arenas.
(PDF)

Text S11 Path integration with vision but no boundary map.
(PDF)

Text S12 Grid fields without vision following arena compression and expansion.
(PDF)

Text S13 Animated example of simulated localization without vision.
(PDF)

Text S14 Utility of modular navigation models.
(PDF)

Video S1 Simulated localization without vision between 0 and 8 minutes.
(AVI)

Video S2 Simulated localization without vision between 40 and 48 minutes.
(AVI)
