# Supplementary figures and images for: Maintaining a Cognitive Map in Darkness: The Need to Fuse Boundary Knowledge with Path Integration
Source: PLoS Comput Biol. 2012 Aug 16;8(8):e1002651. doi: 10.1371/journal.pcbi.1002651 (PMC3420935; doi:10.1371/journal.pcbi.1002651)

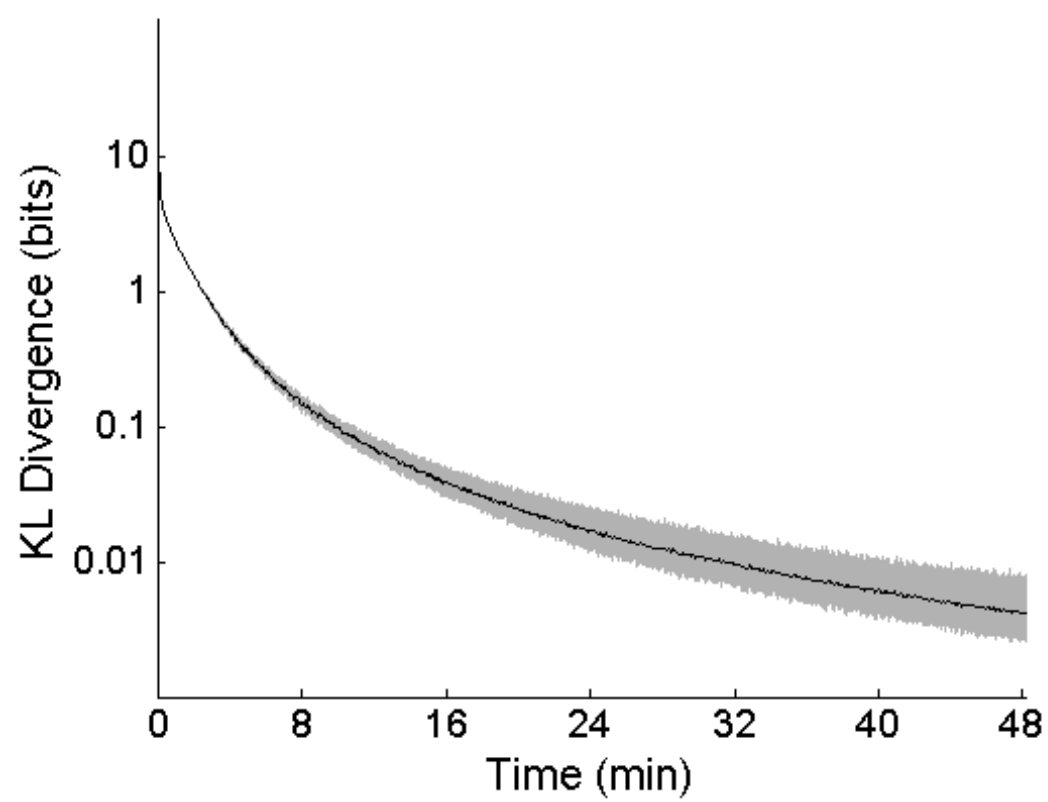

Supplement: Figure S1 — Spatial information content expected from iPI alone. Mean ± s.e.m. of KL divergence of 1,000 trials using HD error model described in Methods, assuming precise linear displacement estimates. See text S5 for further details. (PDF) [file pcbi.1002651.s001.pdf]

A

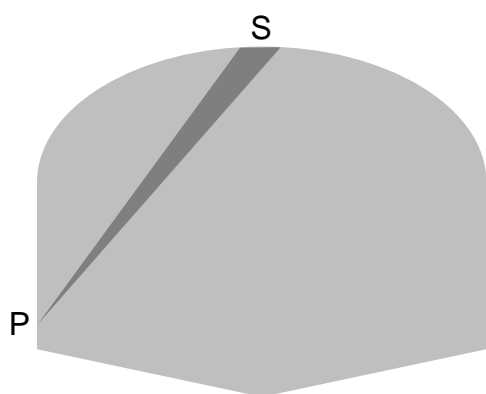

B

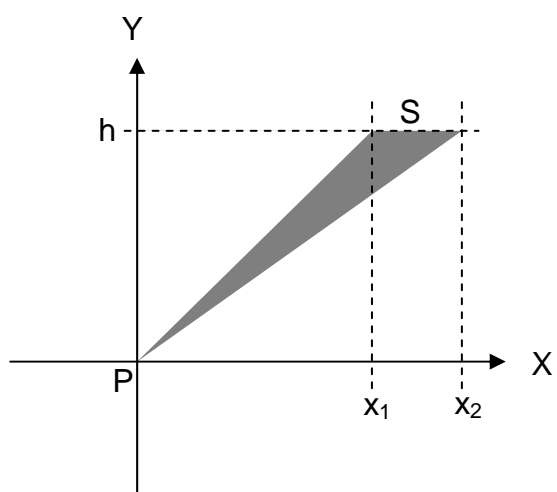

C

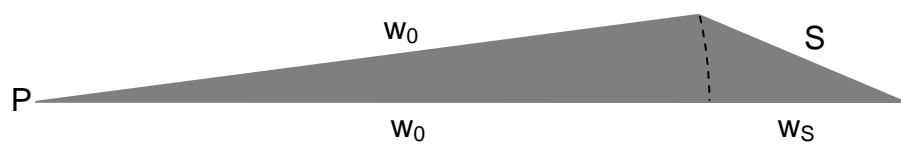

Supplement: Figure S2 — Comparing distances to boundary and interior points of convex shapes. A. Geometric construct showing an arbitrary convex 2D shape (light grey), with a small region (dark grey) indicating all points between point P and segment S along the perimeter. B. Taking the limit as the length of S approaches zero, the dark grey region in (A) is approximated by a scalene triangle. C. An expanded view of the scalene triangle of (B), showing a circular arc of radius w0 (dashed line) centred at P. (PDF) [file pcbi.1002651.s002.pdf]

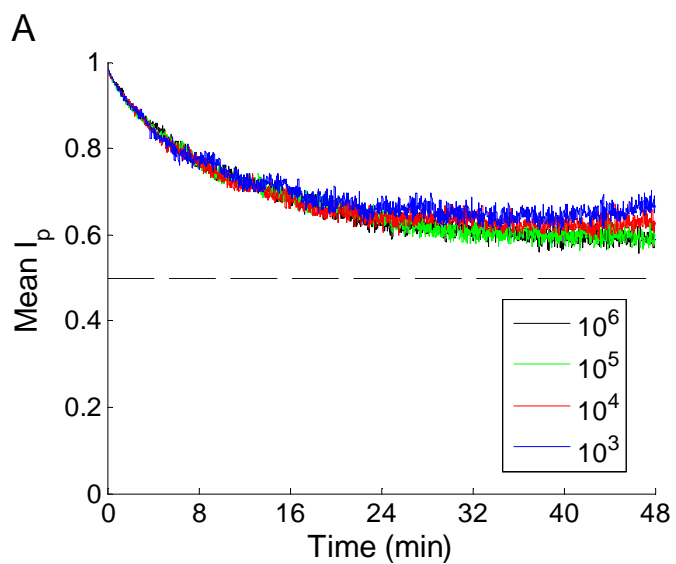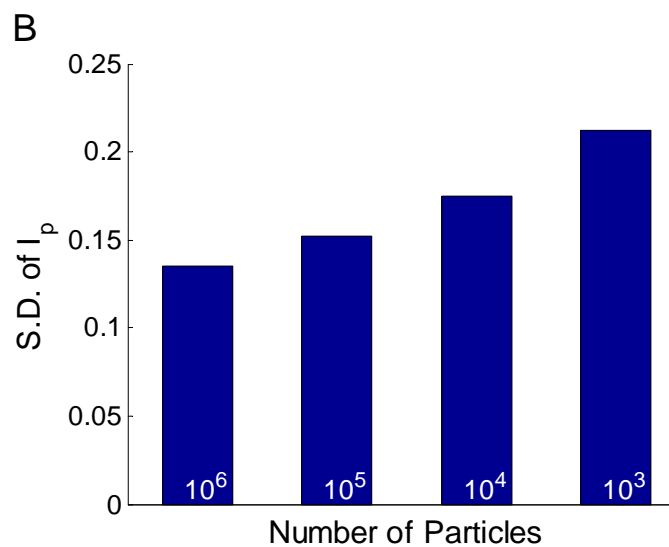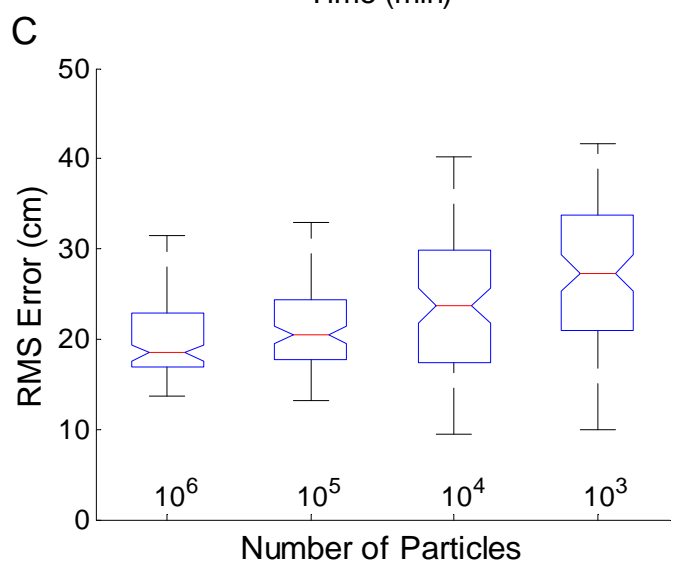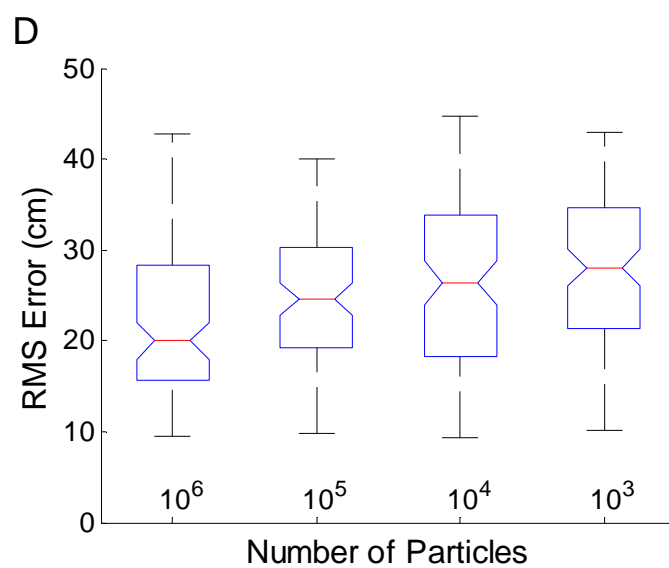

Supplement: Figure S3 — Place stability estimates using different particle cloud sizes. A. The mean place stability index (Ip) is shown for 100 random trials, in a circular arena of 78 cm diameter, using iPI, arena memory and boundary contact information. The particle filter was updated using stochastic universal resampling, with particle cloud populations ranging from 106 to 103. B. One standard deviation of Ip is shown for the last step of the results in A. Notched boxplots are shown for the root-mean-square (RMS) distance error from the true position, using the Cartesian (C) and polar (D) estimates of position, averaged over 48 minutes in darkness for the same trials as A and B. (PDF) [file pcbi.1002651.s003.pdf]

A

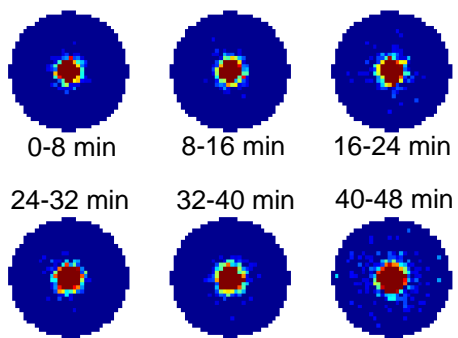

B

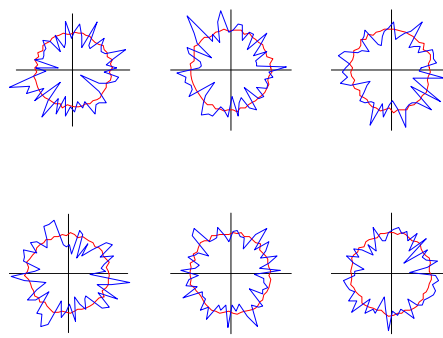

C

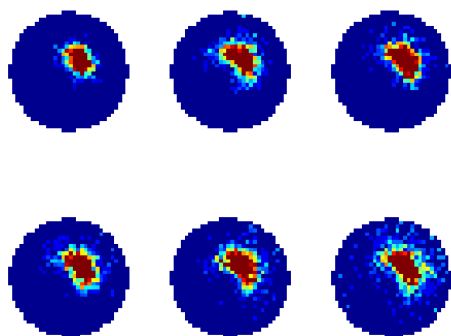

D

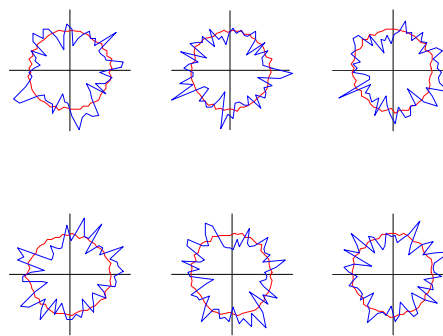

E

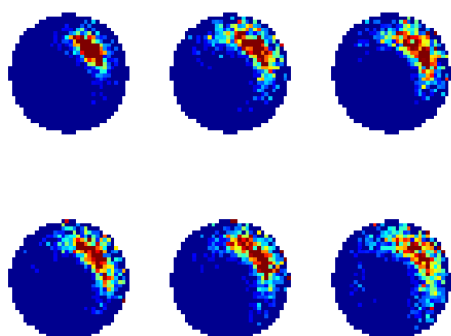

F

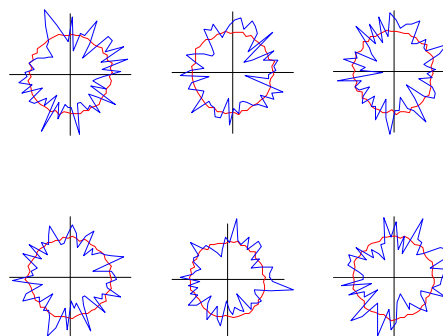

G

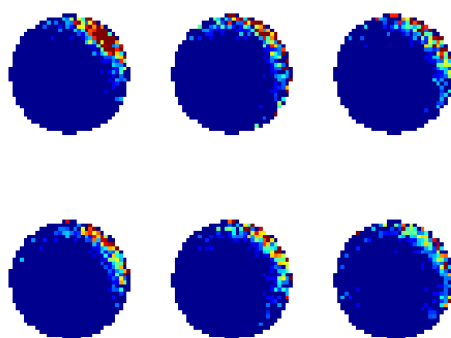

H

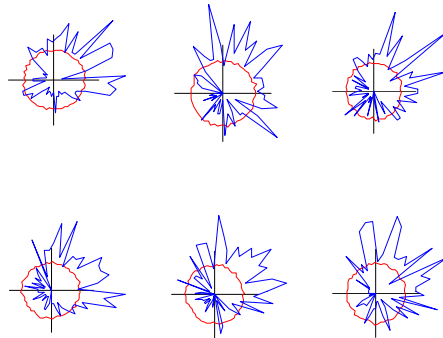

Supplement: Figure S4 — Place stability in darkness using the Cartesian mean of the distributed position estimate. The average of the most stable 10% of place fields (n = 100) are shown in 8 min time windows (A, C, E and G). The ideal locations are 0 cm (A), 10 cm (B), 20 cm (C), and 30 cm (D) from the centre of the arena, along a line at 45° from the horizontal. The corresponding heading distributions during spikes (blue) and over the entire period (red) are also shown (B, D, F and H). For the averaged place fields in each of the 4 locations, there was no significant deviation from the assumption of uniform heading distribution at 0.05 level (Rayleigh's test with Bonferroni correction), and the directional information content was an order of magnitude lower than the spatial information content, typically around or below 0.1 bits/spike (Table S3), consistent with the majority of these fields being pure place representations. All pseudocolour scales used a maximum value of 0.15 spikes/step. The results of G & H are discussed further in Text S9. (PDF) [file pcbi.1002651.s004.pdf]

A

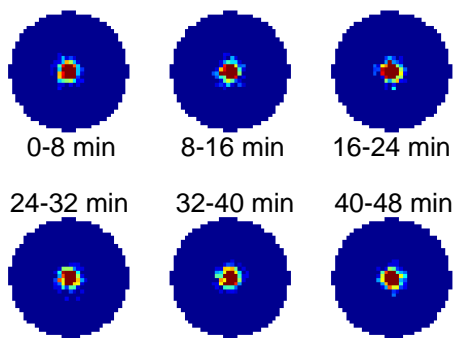

B

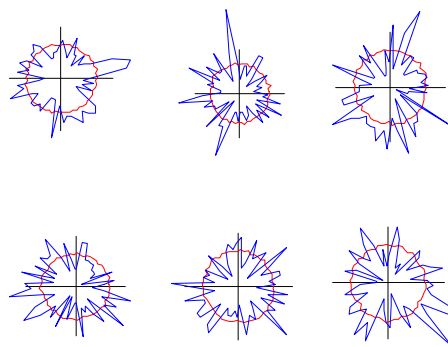

C

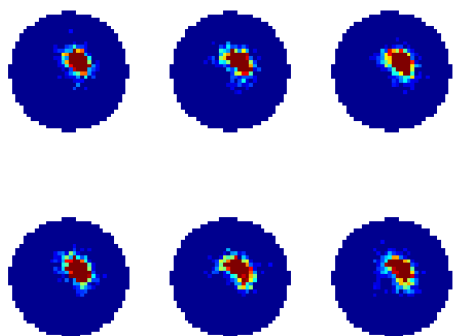

D

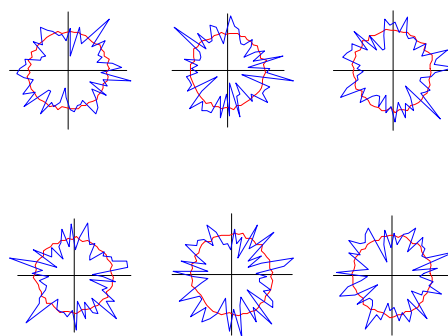

E

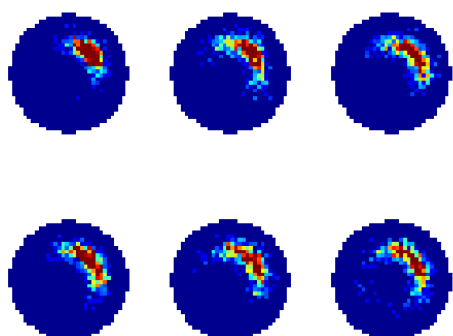

F

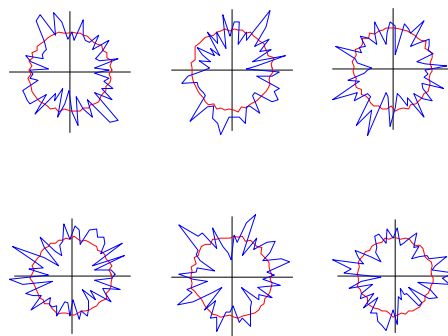

G

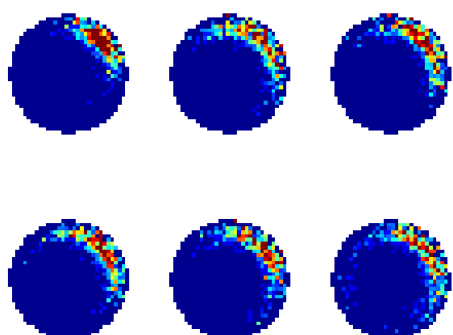

H

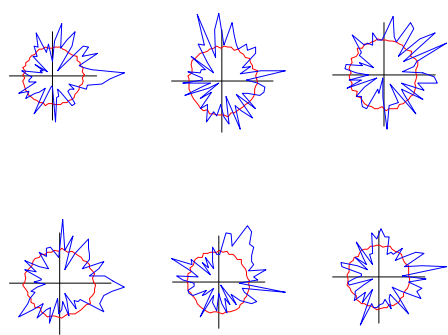

Supplement: Figure S5 — Place stability in darkness using the polar mean of the distributed position estimate. The average of the most stable 10% of place fields (n = 100) are shown in 8 min time windows (A, C, E and G). The ideal locations are 0 cm (A), 10 cm (B), 20 cm (C), and 30 cm (D) from the centre of the arena, along a line at 45° from the horizontal. The corresponding heading distributions during spikes (blue) and over the entire period (red) are also shown (B, D, F and H). For the averaged place fields in each of the 4 locations, there was no significant deviation from the assumption of uniform heading distribution at 0.05 level (Rayleigh's test with Bonferroni correction), and the directional information content was an order of magnitude lower than the spatial information content, typically around or below 0.1 bits/spike (Table S3), consistent with the majority of these fields being pure place representations. All pseudocolour scales used a maximum value of 0.15 spikes/step. The results of G & H are discussed further in Text S9. (PDF) [file pcbi.1002651.s005.pdf]

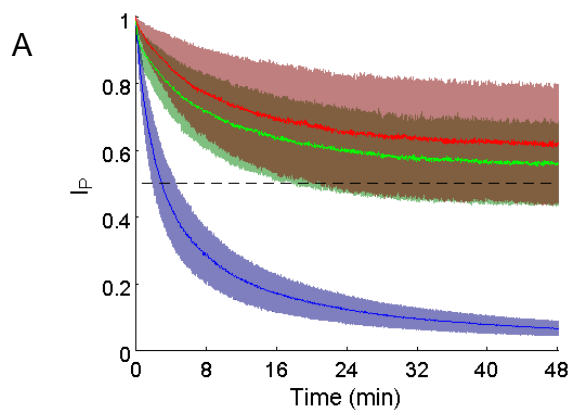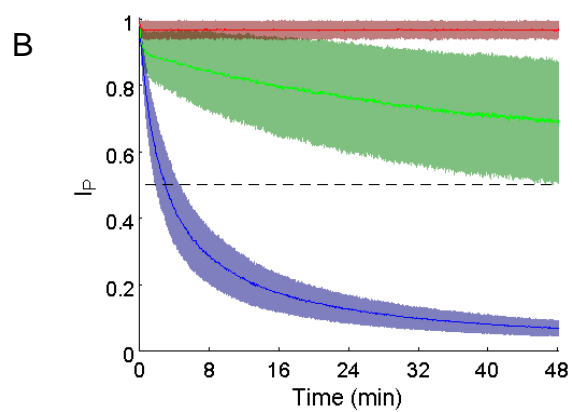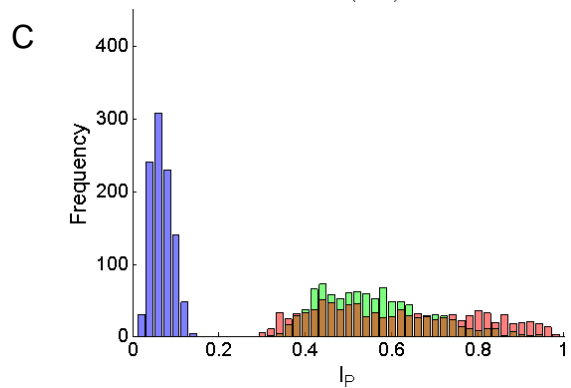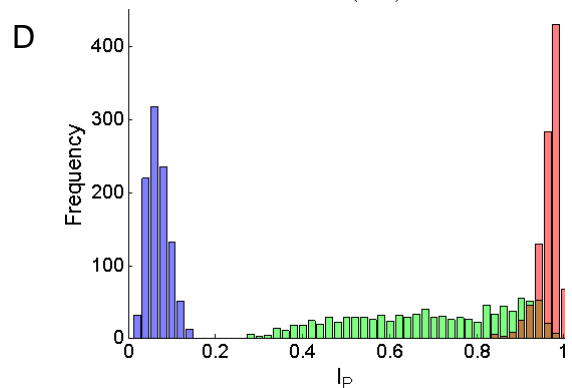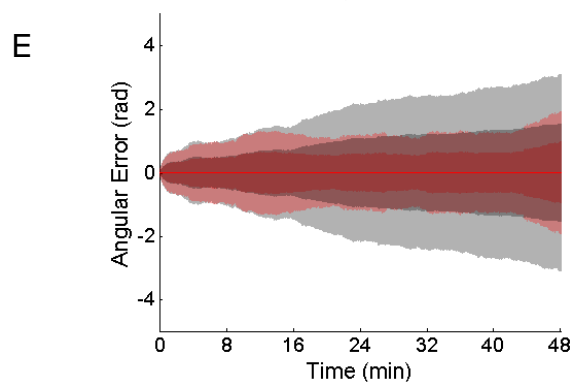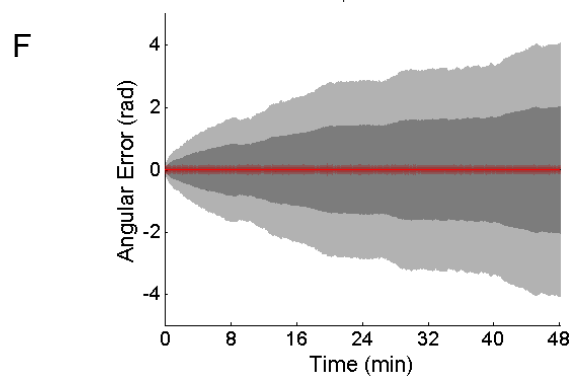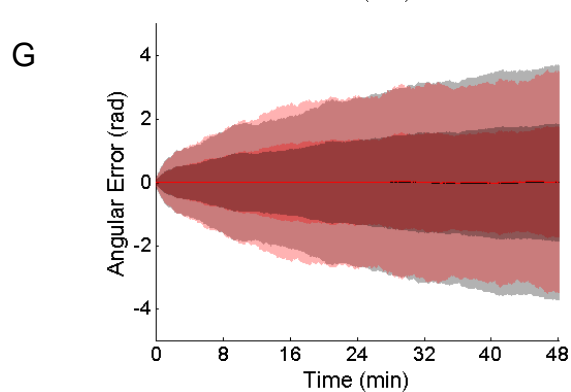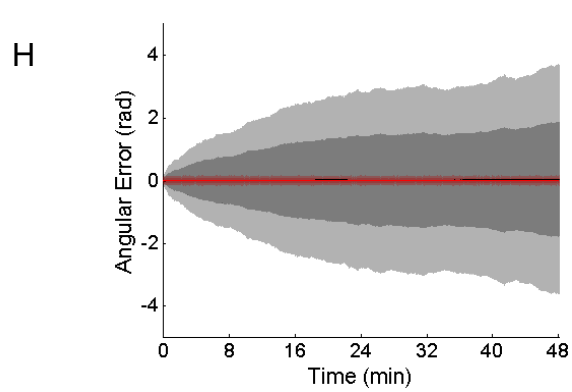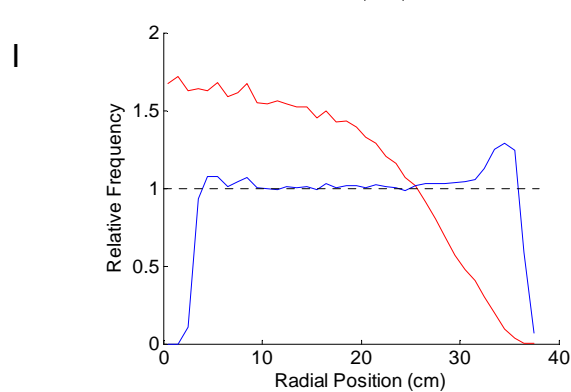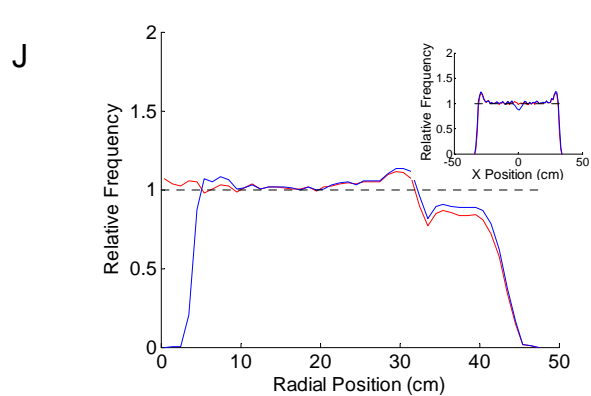

Supplement: Figure S6 — A comparison of localization performance in circular and square arenas without vision. The mean ± s.d. of the place stability index over 48 minutes with vision are shown for using iPI only (blue), iPI and arena memory (green), iPI and arena memory and wall contact information (red) in a circular (A) and square (B) arena. The square arena was the same area as the circular arena which was 76 cm in diameter. The corresponding colour-coded frequency histograms of place stability values at the end of 48 minutes are shown in C (circular arena) and D (square arena). The angular error mean ± 1s.d. and mean ± 2s.d. simulated HD (grey) and particle filter estimate of heading (red) are shown for the circular (E) and square (F) arena, for the top 10% of trials based on place stability index. The angular error mean ± 1s.d. and mean ± 2s.d. simulated HD (grey) and particle filter estimate of heading (red) are shown for the circular (G) and square (H) arena, for a random 10% of trials. For the top 10% trials based on place stability index used to generate place fields in Table S3, the relative frequency distributions of the Cartesian (red) and polar (blue) mean estimates of radial position are shown for the circular (I) and square (J) arena. Each raw count was normalized by dividing by the actual frequency of the simulated rat being in each radial position bin of 1 cm width. The dotted line shows the ideal relative frequency distribution assuming error-free position tracking. The inset shows the relative frequency with respect to X position in the square arena. (PDF) [file pcbi.1002651.s006.pdf]

A

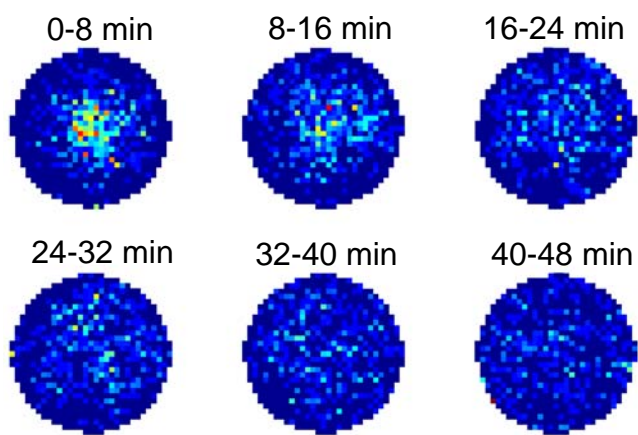

B

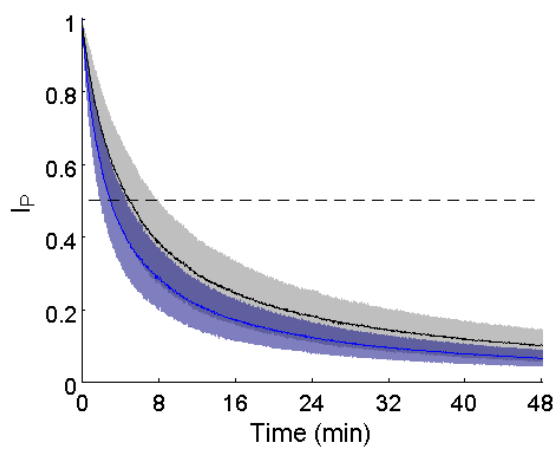

Supplement: Figure S7 — Place fields using allothetic path integration (aPI) only. A. Place fields were generated using aPI only. Fields were centred at (0,0) to maximize spatial information content (see S3). Stable place representations could not be maintained using aPI alone. B. Allothetic PI (grey) led to higher average place stability than iPI (blue). Mean ± s.d. of 1,000 trials are shown for each PI model. In a typical case, the place stability index dropped below chance (dashed line) within 8 minutes using aPI alone (on average in under 5 minutes). (PDF) [file pcbi.1002651.s007.pdf]

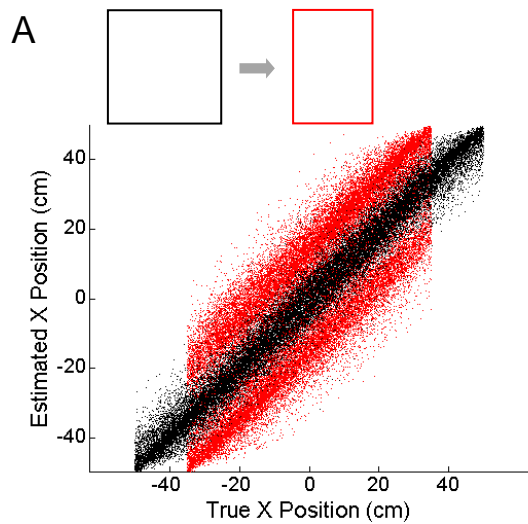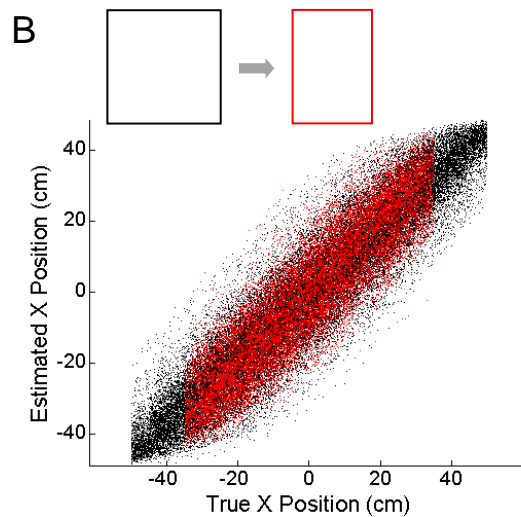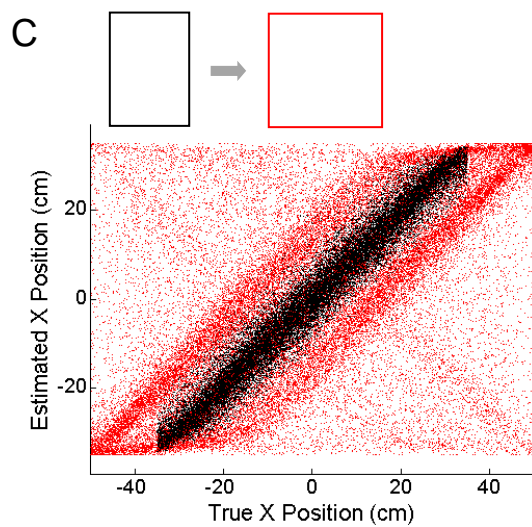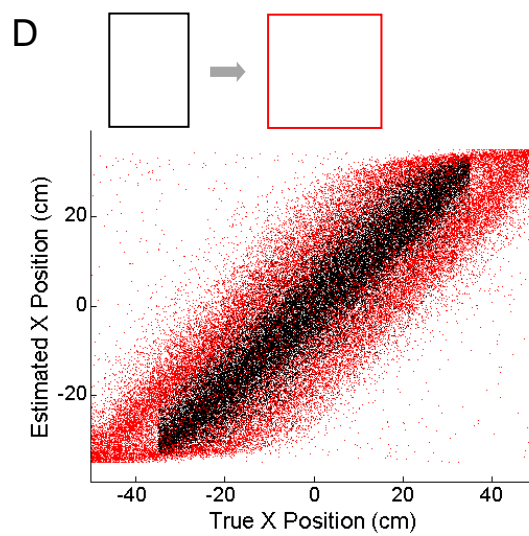

Supplement: Figure S8 — The effect of arena transforms on the estimated position in darkness. The mean particle cloud X position is plotted against the true X position from 10 random trials in each of two reciprocal arena transform simulations, using each of two stochastic resampling methods. The training arena was 100 cm by 100 cm (A & B), and 100 cm by 70 cm (C & D). Examples using both the standard stochastic universal resampling method (A & C) and resampling of heading only (B & D) are included for comparison. The colour-coded original (black) and transformed (red) arenas are shown schematically above each corresponding plot. (PDF) [file pcbi.1002651.s008.pdf]

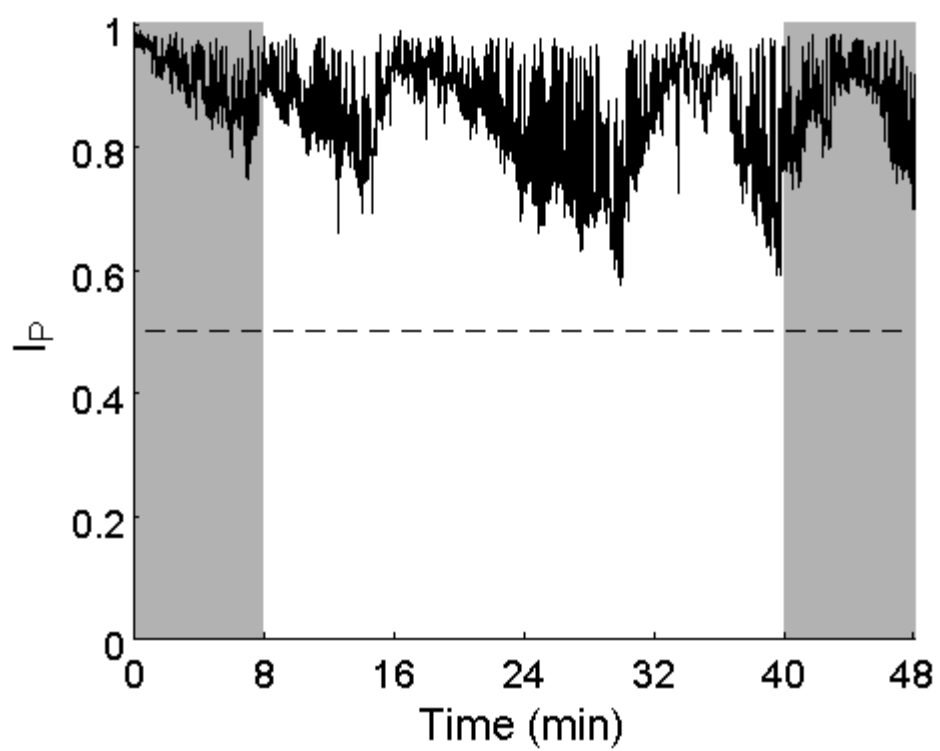

Supplement: Figure S9 — Place stability index of the example described in Text S12. The corresponding particle cloud dynamics are shown in Video S1 and Video S2 (periods indicated in grey). (PDF) [file pcbi.1002651.s009.pdf]
