# Supplementary material for: Maintaining a Cognitive Map in Darkness: The Need to Fuse Boundary Knowledge with Path Integration
Source: PLoS Comput Biol. 2012 Aug 16;8(8):e1002651. doi: 10.1371/journal.pcbi.1002651 (PMC3420935; doi:10.1371/journal.pcbi.1002651)
Supplement: Table S1 — Mean squared distance errors in circular and square arenas, assuming location at the boundary. (PDF) [file pcbi.1002651.s010.pdf]

Table S1 – Mean squared distance errors in circular and square arenas, assuming location at the boundary.

W+	$\left\langle D_0^{\ 2} \mid x, y \right\rangle$	$\left\langle D_P^{\ 2} \mid x, y \right\rangle$
Circular	$3r^2/2$	$2r^2$
Square	$x^2 + y^2 + w^2/6$	$x^2 + y^2 + w^2/3$
