# Supplementary material for: Maintaining a Cognitive Map in Darkness: The Need to Fuse Boundary Knowledge with Path Integration
Source: PLoS Comput Biol. 2012 Aug 16;8(8):e1002651. doi: 10.1371/journal.pcbi.1002651 (PMC3420935; doi:10.1371/journal.pcbi.1002651)
Supplement: Table S2 — Mean place stability index in circular and square arenas. (PDF) [file pcbi.1002651.s011.pdf]

Table S2 – Mean place stability index in circular and square arenas.

Arena Shape	Average Place Stability Index	
	$\langle I_P \mid W+\rangle$	$\langle I_P \mid W-\rangle$
Circular	0.4286	0.5000
Square	0.4275	0.5000
