# Supplementary material for: Maintaining a Cognitive Map in Darkness: The Need to Fuse Boundary Knowledge with Path Integration
Source: PLoS Comput Biol. 2012 Aug 16;8(8):e1002651. doi: 10.1371/journal.pcbi.1002651 (PMC3420935; doi:10.1371/journal.pcbi.1002651)
Supplement: Table S3 — Properties of simulated place fields in a circular arena. (PDF) [file pcbi.1002651.s012.pdf]

Table S3 - Properties of simulated place fields in a circular arena.

Cartesian mean of particle cloud position estimate							
Property	Radial position	Time					
		0-8min	8-16min	16-24min	24-32min	32-40min	40-48min
Spatial information (bits/spike)	0cm	3.674556	3.571258	3.457551	3.392681	3.412098	3.00895
	10cm	3.261239	2.542717	2.621484	2.569233	2.412031	2.020061
	20cm	2.831038	2.057825	2.140235	2.19268	2.077288	1.795725
	30cm	3.335579	3.250581	3.273341	3.233897	3.132384	3.128845
Directional information (bits/spike)	0cm	0.041847	0.038666	0.028708	0.043126	0.023611	0.026777
	10cm	0.034485	0.032113	0.03738	0.040986	0.037506	0.02968
	20cm	0.052981	0.051687	0.062239	0.04443	0.038883	0.055162
	30cm	0.094518	0.240577	0.22094	0.154769	0.319183	0.280091
Spikes	0cm	919	1076	1103	1080	1209	1519
	10cm	890	1079	1091	1128	1174	1287
	20cm	804	932	942	929	952	965
	30cm	432	278	267	251	266	222
R	0cm	1	0.975686	0.971722	0.971464	0.973408	0.958472
	10cm	1	0.916391	0.885312	0.918935	0.901989	0.850538
	20cm	1	0.740511	0.70648	0.719948	0.632756	0.524941
	30cm	1	0.374191	0.462857	0.519611	0.632453	0.388953
R_{max}	0cm	1	0.976796	0.977083	0.971414	0.976373	0.963041
	10cm	1	0.904085	0.884353	0.903194	0.900841	0.852474
	20cm	1	0.782565	0.760137	0.786553	0.751757	0.683094
	30cm	1	0.626135	0.707608	0.695021	0.692706	0.662639
$\Delta\theta_{max}$ (°)	0cm	N/A*					
	10cm	0	-1	0	1	-3	7
	20cm	0	1	0	3	7	-5
	30cm	0	12	8	11	4	0
Coherence	0cm	0.967767	0.975292	0.973894	0.97648	0.980025	0.976664
	10cm	0.963402	0.965041	0.959354	0.956044	0.957557	0.942884
	20cm	0.936281	0.901708	0.879076	0.880516	0.864356	0.846038
	30cm	0.910753	0.566805	0.734283	0.785527	0.764981	0.720978

Polar mean of particle cloud position estimate							
Property	Radial position	Time					
		0-8min	8-16min	16-24min	24-32min	32-40min	40-48min
Spatial information (bits/spike)	0cm	4.024516	4.050488	4.077583	4.029865	4.048992	4.152669
	10cm	3.561757	3.300398	3.249514	3.235175	3.179041	3.074484
	20cm	3.156433	2.549257	2.540526	2.60719	2.56009	2.409524
	30cm	3.128432	2.621022	2.747887	2.702138	2.654352	2.471658
Directional information (bits/spike)	0cm	0.07565	0.132734	0.135035	0.125847	0.097344	0.134659
	10cm	0.036655	0.075184	0.042936	0.057134	0.066016	0.046773
	20cm	0.052016	0.072719	0.056188	0.066963	0.071467	0.062376
	30cm	0.097991	0.104033	0.094762	0.068934	0.106029	0.081208
Spikes	0cm	381	377	352	354	347	398
	10cm	785	746	737	706	693	675
	20cm	671	657	672	697	685	663
	30cm	497	517	531	542	521	545
R	0cm	1	0.941309	0.930032	0.928566	0.951451	0.940139
	10cm	1	0.949512	0.934494	0.937344	0.907602	0.903212
	20cm	1	0.881351	0.841289	0.847701	0.818375	0.802163
	30cm	1	0.617117	0.667643	0.700137	0.735508	0.656524
R_{max}	0cm	1	0.960307	0.938963	0.958638	0.965476	0.959362
	10cm	1	0.94806	0.93989	0.942501	0.912716	0.906243
	20cm	1	0.88132	0.857692	0.858118	0.847544	0.826228
	30cm	1	0.764436	0.806756	0.821722	0.81904	0.726003
$\Delta\theta_{max}$ (°)	0cm	N/A					
	10cm	0	-1	-4	1	-5	-7
	20cm	0	1	12	2	7	11
	30cm	0	-11	3	1	0	-3
Coherence	0cm	0.935026	0.947315	0.954564	0.924772	0.94369	0.960068
	10cm	0.962573	0.95921	0.965403	0.959459	0.962309	0.954075
	20cm	0.939109	0.925364	0.917684	0.924331	0.890945	0.912106
	30cm	0.915909	0.701413	0.858829	0.847617	0.792117	0.80776

*Fields at the center of the arena are approximately radially symmetric so estimates of angular drift have no clear meaning and are due largely to random variations in spike counts.
