# Supplementary material for: Maintaining a Cognitive Map in Darkness: The Need to Fuse Boundary Knowledge with Path Integration
Source: PLoS Comput Biol. 2012 Aug 16;8(8):e1002651. doi: 10.1371/journal.pcbi.1002651 (PMC3420935; doi:10.1371/journal.pcbi.1002651)
Supplement: Table S4 — Properties of simulated place field using allothetic path integration only. (PDF) [file pcbi.1002651.s013.pdf]

Table S4 – Properties of simulated place field using allothetic path integration only.

Property	Time					
	0-8min	8-16min	16-24min	24-32min	32-40min	40-48min
Spatial information*	1.6645	1.4777	1.3667	1.4894	1.5099	1.6002
Directional information*	0.1032	0.0960	0.0968	0.1152	0.1142	0.1548
Spikes	502	505	461	429	386	340
R	1.0000	0.4277	0.1912	0.2397	0.0769	0.0183

*bits/spike
