# Supplementary material for: Maintaining a Cognitive Map in Darkness: The Need to Fuse Boundary Knowledge with Path Integration
Source: PLoS Comput Biol. 2012 Aug 16;8(8):e1002651. doi: 10.1371/journal.pcbi.1002651 (PMC3420935; doi:10.1371/journal.pcbi.1002651)
Supplement: Text S1 — Simulated random trajectories. (PDF) [file pcbi.1002651.s014.pdf]

S1 – Simulated Random Trajectories

Random trajectories were generated stepwise as follows:

1) Change intended walking direction by a random angle $\alpha \sim N\left(0,\sigma_\alpha^2\right)$, where $\sigma_\alpha = 0.5$ rad.
2) Move forward by random step length $l \sim N\left(\mu_l,\sigma_l^2\right)$ cm if within the arena and repeat from step (1), otherwise go to step (3). Estimated step length parameters in darkness $\mu_l = 7$ cm and $\sigma_l = 0.2\mu_l$.
3) Randomly choose one of two options with probability p and $1-p$ respectively: either turn towards the interior of the arena so that the boundary normal bisects the turn angle, or turn randomly according to (1) until the intended step remained within the arena. Return to step (2).

The random angle α determined the tortuosity (the degree of twist) of the trajectory in between boundary contacts. Each trajectory segment was equivalent to a correlated random walk [6] or idiothetic directed walk [8,9]. The composite step (3) ensured that the entire arena was sampled approximately homogeneously so that there was no excessive thigmotactic (wall-following) behaviour. The value of p was chosen to ensure even coverage of the simulated arenas. In all the simulated trajectories in this work, the dwell time density within one step of the perimeter was within ±5% of the rest of the arena. For practical reasons, in real arenas, the robot iRat's trajectories were generated by a slightly different real-time control algorithm whose turn decisions were based on IR proximity readings (Fig 2C).

The rat was assumed to have an average speed of 9cm/s and average stride length of $\mu_l = 7cm$ in darkness. This was based on the walking speed of blind rats in 2D enclosures [42] and the observation that rats take between one and two strides per second on linear tracks [S1, S2]. This gave the estimated number of steps in 8 minutes as $n_8 \approx 617$. Preliminary simulations showed that place stability was relatively insensitive to changes in μ_l but was adversely affected by increasing the average speed. This was due to the fact that the estimate of HD error was time based (Text S2).

All simulations began at the centre of the arena, with initial heading at 0° (to the right of diagrams). The navigation system was updated at the end of each forward step.

S1. Hruska RE, Kennedy S, Silbergeld EK (1979) Quantitative aspects of normal locomotion in rats. Life Sci 25: 171-180.
S2. Berryman ER, Harris RL, Moalli M, Bagi CM (2009) Digigait quantitation of gait dynamics in rat rheumatoid arthritis model. J Musculoskelet Neuronal Interact 9: 89-98.
