# Supplementary material for: Maintaining a Cognitive Map in Darkness: The Need to Fuse Boundary Knowledge with Path Integration
Source: PLoS Comput Biol. 2012 Aug 16;8(8):e1002651. doi: 10.1371/journal.pcbi.1002651 (PMC3420935; doi:10.1371/journal.pcbi.1002651)
Supplement: Text S2 — Head direction error model. (PDF) [file pcbi.1002651.s015.pdf]

S2 – Head Direction Error Model

In darkness, the rodent HD system accrues error whose distribution is approximately bell-shaped and zero-mean [40]. The standard deviation was estimated from the data as follows. Head direction error accumulates at an approximately constant rate [40, 41] which we approximate as a Wiener process. In discrete time steps, the variance per step is $\sigma_\delta{}^2 \approx \sigma_T^2 / n_8$, where n_8 is the total number of steps in 8 minutes (Text S1). Alternatively, the variance per second is $\sigma_t{}^2 \approx \sigma_T{}^2 / 480$.

The HD tuning of individual HD cells in ADN and PoS drifted, in absolute terms, by 23°±6° between the first 2 minutes and last 2 minutes of an 8 minute session without vision [40]. Since each HD cell was recorded from a different trial, we assume that HD tuning across cells are independent, and can therefore be approximated as a normally distributed random variable. Hence the standard deviation $\sigma = \sqrt{\pi/2}\left\langle |\theta - \mu_\theta| \right\rangle$ where $|\theta - \mu_\theta|$ is the absolute angular deviation, θ is the HD tuning, and $\mu_\theta = \langle \theta \rangle$ is the true heading.

For a Wiener process starting at 0, the mean absolute drift averaged over the first two minutes is thus

$$\left\langle |\theta - \mu_\theta| \right\rangle_{0-2\min} = \left\langle |\theta| \right\rangle_{0-2\min} = \frac{\sigma_0}{2}\int_0^2 \sqrt{t}\,dt = \sigma_0 \frac{2^{\frac{3}{2}}}{3} \qquad \text{(S2.1)}$$

where σ_0 is the HD error standard deviation following one minute, assuming ideal alignment with the true direction at time 0. Similarly,

$$\left\langle |\theta| \right\rangle_{6-8\min} = \frac{\sigma_0}{2}\int_6^8 \sqrt{t}\,dt = \sigma_0 \frac{1}{3}\left(8^{\frac{3}{2}} - 6^{\frac{3}{2}}\right) \qquad \text{(S2.2)}$$

And overall, $\left\langle |\theta| \right\rangle_{0-8\min} = \sigma_0\sqrt{8}$, so

$$\frac{\left\langle |\theta| \right\rangle_{0-8\min}}{\left\langle |\theta| \right\rangle_{6-8\min} - \left\langle |\theta| \right\rangle_{0-2\min}} = \frac{8^{\frac{1}{2}}}{\frac{1}{3}\left(8^{\frac{3}{2}} - 6^{\frac{3}{2}}\right) - \frac{1}{3}2^{\frac{3}{2}}} \approx 1.66 \qquad \text{(S2.3)}$$

Hence over 8 minutes, $\sigma_T \approx 23° \times 1.66 \times \sqrt{\pi/2} \approx 48°$. For convenience we approximated this as $\sigma_T^2 \approx \pi^2/16\,rad^2$. This error rate is superimposed onto the true rotation rate (some α per step) of the head.

From [41], in 19 cells of the ADN (control condition), the absolute drift rate was reported to range from 5.1 to 26.6°/min. This was found by comparing the mean change in each ADN HD cell's preferred direction between 0-1 min and 1-2 min, between 1-2 min and 2-3 min, etc. Using the parameters used in the current

simulations (based on [40]), and for a sample size of 19, the 95% CI was found to be 5.1 to 17.0 °/min (mean 10.4°/min) comparable to (Ref 41 - Fig 1B), confirming that the error model used is a reasonable first approximation.

It should be noted that the entire HD system is assumed to drift coherently, e.g., random directional error cannot be reduced significantly by 'cancelling' across multiple HD cells.
