# Supplementary material for: Maintaining a Cognitive Map in Darkness: The Need to Fuse Boundary Knowledge with Path Integration
Source: PLoS Comput Biol. 2012 Aug 16;8(8):e1002651. doi: 10.1371/journal.pcbi.1002651 (PMC3420935; doi:10.1371/journal.pcbi.1002651)
Supplement: Text S3 — Particle filter navigation model. (PDF) [file pcbi.1002651.s016.pdf]

S3 – Particle Filter Navigation Model

In the following sections, we describe the operational details of the particle filter used in the simulations. In essence, a particle cloud is used to approximate a joint probability distribution of the current position and orientation (pose). As the simulated animal moves, noisy sensory information provides an approximation of self motion. Following each step, the pose distribution is updated according to the estimate of self motion, with some extra noise added. The latter accounts for the possibility of errors in the self motion estimate, so that at least some of the independently propagated particles correspond closely to the true pose of the simulated rat. Two pieces of information are used to refine the estimate of position i.e., reducing the spread of the particle cloud. The first is assumed knowledge of the arena geometry, which allows removal of particles which have been propagated to outside of the extent of the arena. The second is wall contact, which allows the remaining particles to be ranked in terms of how well they would account for the current wall contact information (distance and angle relative to nearest wall) given the known arena geometry. The size of the particle cloud is kept constant by removing particles which do not account for sensory data well, and cloning the remainder.

We used a particle filter in preference to a neural navigation model for several reasons. First and foremost, to investigate theoretical issues in the accumulation of error, a particle filter model enables explicit incorporation of exact noise and process parameters. This allows moment by moment access to the error distribution of the ideal position estimate. Secondly, the straightforward operation of a particle filter provides a simple means of boundary contact resetting. Boundary contact information can be used to recalibrate or reset the particle filter's state representation through simple particle culling or reweighting. Neural models such as the RatSLAM navigation model have complex, interacting system dynamics that make setting motion parameters difficult and would require the development of an arbitrary neural unit reactivation scheme [33].

The particle filter approximates the Bayes-optimal distributed conjunctive position and orientation (pose) estimate of the head. The inputs consist of

1) an erroneous HD system which provides approximate angular displacement information, as described (Text S2);
2) an erroneous step size estimate with error $\varepsilon_l \sim N\left(0,\sigma_l^2\right)$ where $\sigma_l = 1.4\,\text{cm}$ matching the true variability in step size (Text S1);
3) an estimate of distance and direction relative to the nearest wall whenever the rat has to make a systematic turn to avoid wall collision. The angular and linear errors (e.g., from whisking) were assumed to be independent of, but the same magnitude as, those of the PI system per step.

Unless otherwise specified, a cloud of 10^4 particles was used to approximate the head pose. Each particle was propagated as an independent correlated random walk with linear displacement $l_{MC} \sim N\left(l, \sigma_l^2\right)$ and angular displacement $\Delta\theta_{MC} \sim N\left(\alpha, \sigma_\delta^2\right)$, where mean angular turn and step length were the erroneous HD and step size estimates described earlier. In simple terms, the particles have random jitter which simulates a range of possible but unknown errors in angular and linear displacement estimates. This process is analogous to a propagation of the probabilistic estimate of head pose according to the cumulative error rates of iPI. The particle cloud may also be considered as an approximation of the prior probability distribution of pose when either arena memory or wall contact information is to be combined.

Unless otherwise specified, it was assumed that the arena boundary size and shape were precisely known. This information was used to update the pose distribution by modifying the particle cloud as follows. At each step, any particle outside of the arena was removed, and a random particle within the arena cloned. This process is equivalent to the multiplication of the likelihood term in a standard formulation of Bayes' theorem. The likelihood distribution here may be considered as a uniform distribution over the area of the arena in memory, and zero everywhere else. Hence particles outside the arena were assigned a likelihood (weight) of zero. The redistribution of points is equivalent to the resampling procedure described below, and may be considered to be analogous to the normalizing denominator whereby the total number of particles (total distribution probability) is preserved.

On contact with an arena wall, the importance of particle *j* was given by the weight

$$w_j = e^{-\left[\frac{\left(d_w - d_j\right)^2}{2\sigma_l^2} + \frac{\left(\omega_w - \omega_j\right)^2}{2\sigma_\delta^2}\right]} \qquad \text{(S3.1)}$$

where d_w and ω_w are the distance and angle respectively to the nearest point on the wall estimated during wall contact, while d_j and ω_j are the distance and angle of particle *j* relative to its nearest point on the wall. This weight is in fact proportional to the conditional probability (likelihood) of the observed wall contact sensory information, given the pose of particle *j*. The proportionality constant was dropped since it was the same for all particles so did not affect the resampling process. For simplicity, error standard deviations were assumed to be available to the navigation system. Such information may be hardwired through natural selection or learnt through experience.

Particle Resampling Process

In particle filter applications, it is necessary to prevent particle weights from becoming degenerate, i.e., becoming extremely large or approaching zero. This occurs frequently in practice as only a few particles remain close to the true pose and are assigned large weights, whereas the majority are poor estimates of true

pose and have diminishingly small weights. Resampling enables particles with large weights to be cloned, so that pose space which match sensory data well is sampled more heavily. At the same time, particles with diminishingly small weights are removed as they are unlikely to provide reasonable estimates of true pose in the future.

In brief, stochastic universal resampling of n particles with weights $w_1, w_2, \ldots, w_n$ is carried out as follows. Divide an interval W_0 into n segments in proportion to the n weights. Divide a second interval of equal length, W_1, into n even segments, and place a particle at the centre of each. These are the new particles. Shift the entire set of new particles by the same random distance such that they remain in W_1. Align W_0 and W_1 in parallel, and assign the pose of particle j in W_0 to any new particle(s) which are within its interval. Using this procedure over a large number of trials, the probability of any particle being cloned is proportional to its weight, normalized by the total number of particles. Given an asymptotically large particle population, the result of stochastic universal resampling approaches an exact Bayesian posterior distribution. Once resampling has been completed, each particle has the same unitary weight. This same procedure may be applied following fusion of any sensory information in which the particle weight changes.

The Particle Cloud Population Size

The main purpose of using a particle filter in this work was to test whether it is sufficient for a drifting HD system to be used to maintain a stable representation of place. It was clear that the most challenging case under consideration was localization in a circular arena (see results for details). Hence, the particle cloud size was chosen to ensure that the performance was sufficiently close to Bayes-optimal in a circular arena, while preserving computational tractability. It can be seen that using 10^4 particles was sufficient to replicate an average place stability index ($\overline{I_p}$) comparable to larger particle populations (Fig S3A) while the variability in I_p was not much larger even after 48 minutes of simulated navigation without vision (Fig S3B). Using 10^4 particles, it was feasible to use a standard desktop computer to simulate a range of arenas and experiments, keeping the cloud size constant, with 1,000 random trials per scenario.

It should be noted that the place stability index was developed to measure both the accuracy and precision of an uncertainty distribution. However, a small particle population is at best a coarse sample of the true uncertainty distribution and cannot maintain low probability pose estimates for an extended period of time. In essence, the full spread of the true uncertainty distribution is lost, which tends to cause an inflation of the I_p value due to an inadequate estimate of the spread of the uncertainty distribution (inversely related to precision), but not an improvement in the accuracy (Fig S3A). In contrast, a single best estimate of position is more directly related to how well the true uncertainty distribution is represented. This effect can be seen in the magnitude of the error of the particle cloud's estimate of true position (Fig S3C & S3D). These results also show that some improvement in the performance of the particle filter may be possible if larger particle populations are used. However, the variation in performance over three orders of magnitude of particle

population was relatively small compared to the differences in performance seen across different simulated arenas and navigational information used.

Using Suboptimal Information

It is worth noting that an exact sensory error model was not critical in demonstrating sufficiency in terms of maintaining place stability without vision. In a series of earlier simulations (data not shown) in both circular and rectangular arenas, the wall contact error standard deviations were assumed to be unavailable, and were instead replaced by $\sigma_d = \overline{|d_w - d_j|}$ and $\sigma_\omega = \overline{|\omega_w - \omega_j|}$ respectively. Resampling was also simplified such that the lower 95% of particles by weight were culled, and the remaining 5% were cloned to repopulate the particle cloud. Each particle’s pose was then aligned with its nearest wall (or wall segment) to match (d_w, ω_w). The motivations for these simulations were twofold. Firstly, it provided proof-of-concept that maintaining a stable representation of place without vision could be achieved even if the true sensory error model was not available. This shows that small departures from Bayes-optimal fusion of information may still be compatible with place stability without vision. Secondly, in the iRat experiments, the error distributions of the three IR range sensors were not known so this suboptimal method was used to update the particle cloud. In the iRat experiments, there were between one and three range estimates during wall contact, and each particle weight was assumed to be the product of the weights of all available sensor readings. The main disadvantage of using a suboptimal particle weight and resampling procedure is that the result does not necessarily approach Bayes-optimal, even if the particle size is extremely large.
