# Supplementary material for: Maintaining a Cognitive Map in Darkness: The Need to Fuse Boundary Knowledge with Path Integration
Source: PLoS Comput Biol. 2012 Aug 16;8(8):e1002651. doi: 10.1371/journal.pcbi.1002651 (PMC3420935; doi:10.1371/journal.pcbi.1002651)
Supplement: Text S4 — Place stability index. (PDF) [file pcbi.1002651.s017.pdf]

S4 – Place Stability Index

For any given arena boundary, $\left\langle D_0^{\,2} \mid x,y \right\rangle$ is the expected squared distance between location (x,y) and points distributed uniformly throughout the arena, and $\left\langle D_P^{\,2} \mid x,y \right\rangle$ is the expected squared distance between (x,y) and current distributed estimate of position. Assuming a boundary landmark module independent of PI, the best current estimate of position is represented either by points distributed uniformly along the perimeter (wall contact, denoted $W+$), or by points distributed uniformly over the entire arena (no wall contact, denoted $W-$). Although the wall contact zone is considered as an infinitesimally thin line along the perimeter, in reality there is a finite range within which wall contact occurs. However, for any arena which is much larger than the contact range, there is negligible effect on the results.

For circular and rectangular arenas, it can be shown that

$$\left\langle D_0^{\,2} \mid x,y \right\rangle_{circ} = x^2 + y^2 + r^2/2 \tag{S4.1}$$

$$\left\langle D_0^{\,2} \mid x,y \right\rangle_{rect} = x^2 + y^2 + \frac{w^2 + h^2}{12} \tag{S4.2}$$

When (x,y) is along the boundary,

$$\left\langle D_P^{\,2} \mid x,y \right\rangle_{circ} = 2r^2 \tag{S4.3}$$

$$\left\langle D_P^{\,2} \mid x,y \right\rangle_{rect} = x^2 + y^2 + \frac{\left(w+h\right)^2}{12} \tag{S4.4}$$

where w and h are the width and height of the rectangle respectively (width along the x-axis, and height along the y-axis).

At any true location (x,y), the place stability index is defined as:

$$I_P\left(x,y\right) = \frac{\left\langle D_0^{\,2} \mid x,y \right\rangle}{\left\langle D_0^{\,2} \mid x,y \right\rangle + \left\langle D_p^{\,2} \mid x,y \right\rangle} \tag{S4.5}$$

Assuming a uniform coverage of the entire arena, including the boundary, the expected place stability index when not in contact with the boundary is:

$$\left\langle I_P \mid W- \right\rangle = \left\langle \frac{\left\langle D_0^{\,2} \mid x,y \right\rangle}{\left\langle D_0^{\,2} \mid x,y \right\rangle + \left\langle D_0^{\,2} \mid x,y \right\rangle} \right\rangle_{x,y} = \left\langle \frac{1}{2} \right\rangle = \frac{1}{2} \tag{S4.6}$$

where $\left\langle \cdot \right\rangle_{x,y}$ denotes the expectation over all traversed positions. When in contact with the boundary (i.e. somewhere along the perimeter line), the expected place stability index is:

$$\left\langle I_P \mid W+\right\rangle = \left\langle \frac{\left\langle D_0^{\,2} \mid x,y\right\rangle}{\left\langle D_0^{\,2} \mid x,y\right\rangle + \left\langle D_P^{\,2} \mid x,y\right\rangle} \right\rangle_{x,y} \qquad \text{(S4.7)}$$

For circular arenas, $W+$ implies $x^2+y^2=r^2$ while for square arenas, $W+$ implies $x=\pm w/2$ and/or $y=\pm w/2$. Hence it is straightforward to find the mean squared distance from any boundary location to either all boundary points or to all arena points (Table S1).

Except in circular arenas, the place stability index varies with the true location along the perimeter. For instance, in a square arena, the index is highest when the true location is at one corner, and lowest at the midpoint along one edge.

For circular arenas, the average place stability index is thus constant

$$\left\langle I_P \mid W+\right\rangle = I_P \mid W+ = \frac{3r^2/2}{3r^2/2+2r^2} = \frac{3}{7} \qquad \text{(S4.8)}$$

For square arenas, it can be shown that

$$\left\langle I_P \mid W+\right\rangle = \left\langle \frac{x^2+y^2+w^2/6}{2x^2+2y^2+w^2/2} \right\rangle_{x,y} = \frac{1}{2} - \frac{1}{6\sqrt{2}} \tan^{-1}\left(\frac{1}{\sqrt{2}}\right) \qquad \text{(S4.9)}$$

Thus, even assuming ideal arena contact information, and known sampling distribution of the arena (assumed here to be uniform), the place stability index does not exceed chance level (0.5). In fact, due to the geometric properties of convex shapes such as squares and circles (see Text S7 for further details), the place stability index is always below 0.5 if the posterior positional distribution is used as the distributed representation of current position following wall contact (summarized in Table S2). It is worth noting that these indices are independent of arena size.

Due to the fact that the simulated trajectories sampled the arenas approximately uniformly, the value $\left\langle D_0^{\,2} \mid x,y\right\rangle$ was calculated assuming a uniform distribution of spatial sampling throughout the arena. In practice, the place stability index I_P can be calculated using a trajectory-specific estimate, i.e.,

$$\left\langle D_0^{\,2} \mid x,y\right\rangle \approx \frac{1}{n}\sum_{k=1}^{n} \left(x_k - x\right)^2 + \left(y_k - x\right)^2 \qquad \text{(S4.10)}$$

where n is the number of sample positions along the entire trajectory. Note however that this type of trajectory-specific estimate can produce biased results if that particular trajectory is atypical. Furthermore, trajectory-specific estimates of I_P should not be directly compared unless the spatial sampling of the trajectories are very similar.

Derivations

The place stability index is defined as:

$$I_P(x,y)=\frac{\langle {D_0}^2 \mid x,y\rangle}{\langle {D_0}^2 \mid x,y\rangle+\langle {D_p}^2 \mid x,y\rangle}$$

The Null Distribution

From elementary statistics, it can be shown that for any probability density function $f(x,y)$

$$\langle {D_0}^2 \mid x_0,y_0\rangle = {x_0}^2+{y_0}^2+V(X)+V(Y)$$

where D_0 denotes the distance between a point and (x_0,y_0) and $V(X)$, $V(Y)$ denote the variance of the X and Y positions of the distribution of points respectively.

The expected squared distance from (x_0,y_0) to a uniform distribution of points (the null distribution) can be found by evaluating $V(X)$ and $V(Y)$ for the corresponding arena shape. Next we consider a uniform distribution points in arenas centered at $(0,0)$.

For a circular arena of unit radius,

$$V(X)=\langle X^2\rangle=\frac{2}{\pi}\int_{-1}^{1}x^2\sqrt{1-x^2}\,dx$$
$$=\frac{1}{4\pi}\left[x\sqrt{1-x^2}\left(2x^2-1\right)+\sin^{-1}(x)\right]_{-1}^{1}=\frac{1}{4}$$

Then for a circle of radius r, $V(X)=V(Y)=r^2/4$. Hence $\langle {D_0}^2 \mid x_0,y_0\rangle_{circ}={x_0}^2+{y_0}^2+r^2/2$.

For a rectangular arena,

$$V(X)=\frac{1}{w}\int_{-\frac{w}{2}}^{+\frac{w}{2}}x^2dx=\left.\frac{x^3}{3w}\right|_{-\frac{w}{2}}^{+\frac{w}{2}}=\frac{w^2}{12}$$

Similarly, $V(Y)=h^2/12$. Hence $\langle {D_0}^2 \mid x_0,y_0\rangle_{rect}={x_0}^2+{y_0}^2+\left(w^2+h^2\right)/12$.

Boundary Contact Information

Note that when whiskers are not in contact, i.e. W-, the animal is not at the boundary and the best distributed estimate of position is a uniform distribution over the arena. This is the same as the null condition, so $\langle I_P(W-)\rangle = I_P(W-) = 1/2$.

Circular arena

At the boundary of a circular arena, $x_0^2 + y_0^2 = r^2$ so

$$\langle D_0^2 \mid W+\rangle_{circ} = 3r^2/2$$

Next we find the expected squared distance from one boundary point to a uniform distribution of boundary points. The average squared distance between a point on a circle and every other point on that circle can be found using the following construction

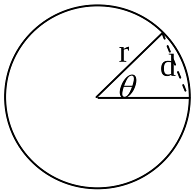

It is straightforward to show that the squared distance given angle θ

$$d_p^2 = r^2\left((1-\cos\theta)^2 + \sin^2\theta\right) = 2r^2(1-\cos\theta)$$

So the expected squared distance over a uniform distribution of points at the perimeter is

$$\langle D_p^2 \mid W+\rangle = \frac{2r^2}{\pi}\int_0^\pi (1-\cos\theta)\,d\theta = \frac{2r^2}{\pi}(\theta - \sin\theta)\Big|_0^\pi = 2r^2$$

Therefore the place stability index is

$$\langle I_P(W+)\rangle = I_P(W+) = \frac{3r^2/2}{3r^2/2 + 2r^2} = \frac{3}{7}$$

Rectangular Arena

At the boundary of a rectangular arena, either $|x_0| = w/2$ or $|y_0| = h/2$ or both. Integrating over the horizontal and vertical edges of the rectangle,

$$\left\langle D_0^{\,2} \mid W+\right\rangle_{rect} = \frac{2}{w+h}\left[\int_0^{w/2}\left(x^2+\left(\frac{h}{2}\right)^2+\frac{w^2+h^2}{12}\right)dx+\int_0^{h/2}\left(y^2+\left(\frac{w}{2}\right)^2+\frac{w^2+h^2}{12}\right)dy\right]$$

$$=\frac{2}{w+h}\left\{\left[\frac{x^3}{3}+\left(\frac{h^2}{3}+\frac{w^2}{12}\right)x\right]_0^{w/2}+\left[\frac{y^3}{3}+\left(\frac{w^2}{3}+\frac{h^2}{12}\right)y\right]_0^{h/2}\right\}$$

$$=\frac{2}{w+h}\left(\frac{w^3}{24}+\frac{wh^2}{6}+\frac{w^3}{24}+\frac{h^3}{24}+\frac{hw^2}{6}+\frac{h^3}{24}\right)$$

$$=\frac{1}{12(w+h)}\left(w^3+2wh^2+h^3+2hw^2\right)$$

$$=\left(w^2+wh+h^2\right)\Big/6$$

The expected squared distance between $(0,k)$ and points distributed uniformly along the line segment $(0,0)$ to $(m,0)$ is:

$$\left\langle D^2\right\rangle=\frac{1}{m}\int_0^m\left(z^2+k^2\right)dz=\frac{m^2}{3}+k^2$$

For convenience, the points along the perimeter of a rectangle are divided into eight segments:

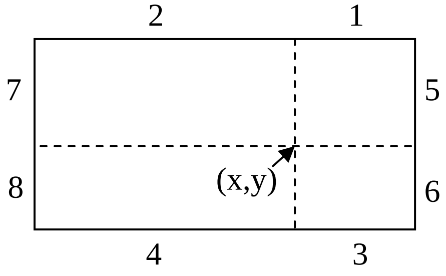

The mean squared distance to each segment is straightforward. Weighting by the length of each segment, the expected squared distance between (x,y) and uniformly distributed points along a rectangular perimeter is thus

$$\left\langle D_P^{\,2} \mid x,y\right\rangle=\frac{1}{2(w+h)}\left[\begin{array}{l}\left(\left(\frac{h}{2}-y\right)^2+\frac{1}{3}\left(\frac{w}{2}-x\right)^2\right)\left(\frac{w}{2}-x\right)+\left(\left(\frac{h}{2}-y\right)^2+\frac{1}{3}\left(\frac{w}{2}+x\right)^2\right)\left(\frac{w}{2}+x\right)\\+\left(\left(\frac{h}{2}+y\right)^2+\frac{1}{3}\left(\frac{w}{2}-x\right)^2\right)\left(\frac{w}{2}-x\right)+\left(\left(\frac{h}{2}+y\right)^2+\frac{1}{3}\left(\frac{w}{2}+x\right)^2\right)\left(\frac{w}{2}+x\right)\\+\left(\left(\frac{w}{2}-x\right)^2+\frac{1}{3}\left(\frac{h}{2}-y\right)^2\right)\left(\frac{h}{2}-y\right)+\left(\left(\frac{w}{2}-x\right)^2+\frac{1}{3}\left(\frac{h}{2}+y\right)^2\right)\left(\frac{h}{2}+y\right)\\+\left(\left(\frac{w}{2}+x\right)^2+\frac{1}{3}\left(\frac{h}{2}-y\right)^2\right)\left(\frac{h}{2}-y\right)+\left(\left(\frac{w}{2}+x\right)^2+\frac{1}{3}\left(\frac{h}{2}+y\right)^2\right)\left(\frac{h}{2}+y\right)\end{array}\right]$$

$$=\cdots=x^2+y^2+\frac{(w+h)^2}{12}$$

Hence the expected squared distance between 2 randomly chosen points from a uniform distribution along the perimeter (which is equivalent to W+) is:

$$\left\langle D_p^{\,2} \mid W+ \right\rangle = \frac{2}{w+h}\left[\int_0^{w/2}\left(x^2+\left(\frac{h}{2}\right)^2+\frac{(w+h)^2}{12}\right)dx+\int_0^{h/2}\left(y^2+\left(\frac{w}{2}\right)^2+\frac{(w+h)^2}{12}\right)dy\right]$$

$$=\cdots=\frac{(w+h)^2}{6}$$

Assuming no information about the position along the boundary, the place stability index when in contact with a rectangular arena is thus given by

$$I_P(W+) = \frac{x^2+y^2+\left(w^2+h^2\right)/12}{x^2+y^2+\left(w^2+h^2\right)/12+x^2+y^2+(w+h)^2/12}$$

For a square arena, $5/12 \le I_p(W+) \le 4/9$. For uniformly distributed visits to the perimeter,

$$\left\langle I_P(W+)\right\rangle = \frac{2}{w}\int_0^{w/2}\frac{x^2+w^2/4+w^2/6}{x^2+w^2/4+w^2/6+x^2+w^2/4+w^2/3}dx$$

$$=\frac{2}{w}\int_0^{w/2}\frac{x^2+5w^2/12}{2x^2+w^2}dx=\frac{2}{w}\left[\frac{\left(5w^2/6-w^2\right)\tan^{-1}\left(\sqrt{2}\,\frac{x}{w}\right)}{w2\sqrt{2}}+\frac{x}{2}\right]_0^{w/2}$$

$$=\frac{2}{w}\left[\frac{-w\tan^{-1}\left(\sqrt{2}\,\frac{x}{w}\right)}{12\sqrt{2}}+\frac{x}{2}\right]_0^{w/2} \quad =\frac{1}{2}-\frac{1}{6\sqrt{2}}\tan^{-1}\left(\frac{1}{\sqrt{2}}\right)$$
