# Supplementary material for: Maintaining a Cognitive Map in Darkness: The Need to Fuse Boundary Knowledge with Path Integration
Source: PLoS Comput Biol. 2012 Aug 16;8(8):e1002651. doi: 10.1371/journal.pcbi.1002651 (PMC3420935; doi:10.1371/journal.pcbi.1002651)
Supplement: Text S5 — Robot iRat experiment. (PDF) [file pcbi.1002651.s018.pdf]

S5 – Robot iRat Experiment

The Intelligent Rat Animat Technology robot (iRat) [52,53] was used for experiments in real arenas (Fig 2A). The iRat's IR sensors are Sharp GP2Y0A41SK0F which gave a non-linear analogue output across a 0.04 - 0.4 m range. Each IR sensor was calibrated using known distances to generate a lookup table for conversion to metric distance. The accuracy of distance estimates depend on minor variations in wall surface reflectance.

The iRat uses a differential wheel drive system for locomotion. The robot receives self-motion feedback on the velocity of its wheels via encoders mounted on the motors. The encoders give 2048 quadrature counts per motor revolution which through a 5:1 gearbox gives 10240 count per wheel revolution which, given a wheel circumference of 0.176 m, means a resolution of 0.017 mm. Wheel slippage causes odometric error which affects both linear and angular displacement estimates in a statistically interdependent way. However, for consistency with computer simulations, we modelled all iRat errors (odometry and IR) as independent Gaussian distributions, fitted empirically to the data.

Movement Algorithm of the iRat

For this study, the iRat was programmed to move, stop, and then log its sensor readings in a repeating sequence as shown in Fig 2C. This loop was repeated for 1500 times at a 5Hz sensor rate resulting in an approximately 5 minute experiment.

Tracking the iRat

The iRat's true pose was tracked using an automated tracking system comprising a web camera mounted directly above the arena. During each experiment, two brightly but differently coloured circle markers were placed on top of the iRat along its rostrocaudal axis (markers not shown in Fig 2A). The markers were at equal distance from the center of rotation of the iRat. The marker positions were found by an automated algorithm which uses standard morphological operations to find the circle centers.

The experiment was performed in a 1 m × 1 m arena. The arena is the same height as the iRat and is a plastic garden edging material. This corresponds to 92 × 92 camera pixels giving a resolution is 0.011 m/pixel. Since many pixels are averaged to find the centers of the circular markers, the pose of the iRat can be estimated at sub pixel precision. However, due to minor variations in lighting, the number of pixels remaining above threshold for each marker may change, with minor effects on accuracy.

Simplified Error Model

Odometric errors were evidently more complex than independent angular and linear displacement errors. There was a clear angular bias (Fig 8B red line) which was not accounted for by the particle filter model. It is likely that localization may be improved further by using a more detailed locomotion error model [9].

A simplified error model was chosen to demonstrate the robustness of the algorithm used. Linear and angular errors were modeled as zero-mean independent Gaussian random variables, whose variances were estimated from a single trial run. Due to the presence of systematic bias, the random error estimate was an overestimation.

We did not supply IR range sensor errors to the particle filter model. Instead, a simple particle filter variant was used to handle particle culling and resampling (Text S3).
