# Supplementary material for: Maintaining a Cognitive Map in Darkness: The Need to Fuse Boundary Knowledge with Path Integration
Source: PLoS Comput Biol. 2012 Aug 16;8(8):e1002651. doi: 10.1371/journal.pcbi.1002651 (PMC3420935; doi:10.1371/journal.pcbi.1002651)
Supplement: Text S6 — Estimating the expected spatial information using iPI only. (PDF) [file pcbi.1002651.s019.pdf]

S6 – Estimating the Expected Spatial Information Using iPI Only

We modelled the uncertainty distribution as a truncated, circularly symmetric bivariate Gaussian distribution in the centre of a circular arena of radius $r_{\max}$. The radial density function (truncated by the arena boundary) is given by

$$p(r) = \frac{re^{-\frac{r^2}{2\sigma^2}}}{\sigma^2\left(1-e^{-\frac{r_{\max}{}^2}{2\sigma^2}}\right)} \tag{S6.1}$$

The variance, σ^2, was estimated from the particle cloud during 1,000 iPI-only simulations. In a uniformly sample circular arena, the radial density function of spatial sampling is given by

$$q(r) = \frac{2r}{r_{\max}{}^2} \tag{S6.2}$$

The KL-divergence between the truncated Gaussian, $p(r)$, and uniform density function, $q(r)$, gives the spatial information in the continuum limit [49].

$$\begin{aligned} D_{KL}(P\,\|\,Q) &= \int_0^{r_{\max}} p(r)\log_2\left(\frac{p(r)}{q(r)}\right)dr \\ &= \left(\frac{r_{\max}{}^2}{2\sigma_p{}^2\left(e^{\frac{r_{\max}{}^2}{2\sigma_p{}^2}}-1\right)}-1\right)\log_2 e + \log_2\left(\frac{r_{\max}{}^2}{2\sigma_p{}^2\left(1-e^{-\frac{r_{\max}{}^2}{2\sigma_p{}^2}}\right)}\right) \end{aligned} \tag{S6.3}$$

The KL-divergence should be considered as an upper limit of spatial information for two reasons. Firstly, it ignores any systematic error between the true position and the estimated mean position.

Secondly, each particle filter simulation provides a distributed position estimate following each step during iPI. In the limit, the particle distribution represents all possible true positions of the rat given an iPI input history. Therefore, the spatial specificity of any neural representation is limited by the uncertainty inherent in the distributed position estimate, irrespective of any errors incorporated during neural processing.

Fig S1 shows the KL divergence over 48 minutes of pure iPI, using the random trajectory model described in Methods, in a circular arena of 76cm diameter. The position estimate variance $\sigma_p{}^2$ was taken as the average of the particle cloud variances along the X and Y directions.
