# Supplementary material for: Maintaining a Cognitive Map in Darkness: The Need to Fuse Boundary Knowledge with Path Integration
Source: PLoS Comput Biol. 2012 Aug 16;8(8):e1002651. doi: 10.1371/journal.pcbi.1002651 (PMC3420935; doi:10.1371/journal.pcbi.1002651)
Supplement: Text S7 — Mean squared distance and general distance measures to convex boundaries. (PDF) [file pcbi.1002651.s020.pdf]

S7 – Mean Squared Distance and General Distance Measures to Convex Boundaries

Assuming the rat has acquired a perfect geometric representation of the arena boundary, its position estimate is always bounded. In the absence of visual and olfactory cues, a homogeneous boundary does not allow the rat to localize to a precise location. However, when in contact with the boundary, the rat's navigation system could theoretically localize to the boundary. We now consider whether localizing to the boundary reduces the error in estimation of current position.

The estimation error is defined as the mean squared distance between the current position and the positional distribution of current position, the latter being the estimate of current position.

As the length of segment S approaches zero, the mean squared distance between point P and a uniform distribution of points along boundary segment S (Fig S2A, S2B) is

$$\left\langle D^2 \right\rangle_S = \frac{1}{x_2 - x_1} \int_{x_1}^{x_2} \left(h^2 + x^2 \right) dx = h^2 + \frac{{x_2}^2 + x_1 x_2 + {x_2}^2}{3} \qquad \text{(S7.1)}$$

Similarly, the mean squared distance between P and a uniform distribution of points in region A is

$$\left\langle D^2 \right\rangle_A = \frac{2}{h\left(x_2 - x_1\right)} \int_0^h dy \int_{\frac{yx_1}{h}}^{\frac{yx_2}{h}} \left(y^2 + x^2 \right) dx = \frac{h^2}{2} + \frac{{x_2}^2 + x_1 x_2 + {x_1}^2}{6} = \frac{\left\langle D^2 \right\rangle_S}{2} \qquad \text{(S7.2)}$$

From any point along a convex 2D arena boundary, it is possible to divide the bounded region into an arbitrarily large number of subregions A_1 to A_n whose corresponding boundary segments S_1 to S_n constitute the entire boundary. Since for every subregion *i*, $\left\langle D^2 \right\rangle_{S_i} > \left\langle D^2 \right\rangle_{A_i}$, then for the entire bounded region, $\left\langle D^2 \right\rangle_{S_{Total}} > \left\langle D^2 \right\rangle_{A_{Total}}$ where S_{Total} denotes the entire perimeter and A_{Total} denotes the entire area. In other words, a totally random guess of current position has a smaller mean squared error than a random guess along the perimeter, even when the true position is actually along the perimeter. This result applies to all convex boundaries including circular and rectangular boundaries. For example, given a circular boundary of radius r_0

$$\left\langle D^2 \right\rangle_{S_{Total}} = \frac{1}{\pi} \int_0^{\pi} \left\langle D^2 \right\rangle_S d\theta = \frac{1}{\pi} \int_0^{\pi} \left(2 r_0 \sin\frac{\theta}{2} \right)^2 d\theta = 2{r_0}^2 \qquad \text{(S7.3)}$$

and

$$\begin{aligned} \left\langle D^2 \right\rangle_{A_{Total}} &= \int_0^{\pi} f_A(\theta) \left\langle D^2 \right\rangle_A d\theta = \int_0^{\pi} f_A(\theta) \frac{\left\langle D^2 \right\rangle_S}{2} d\theta \\ &= \frac{2}{\pi {r_0}^2} \int_0^{\pi} \left(r_0 \sin\frac{\theta}{2} \right)^2 \frac{\left(2 r_0 \sin\frac{\theta}{2} \right)^2}{2} d\theta = \frac{3{r_0}^2}{2} \end{aligned} \qquad \text{(S7.4)}$$

Hence for a circular boundary, $\left\langle D^2 \right\rangle_{S_{Total}} = \tfrac{4}{3}\left\langle D^2 \right\rangle_{A_{Total}}$.

This result can be generalized further. In the limit as $s/w_0 \to 0$ (see Fig S2C), we can write the radial density function of all points within the region, and of all points along line segment S (where the length of S is denoted s). The limit density functions are

$$f_A(D) = \begin{cases} \dfrac{2}{w_0 + w_S}\left(\dfrac{D}{w_0}\right) & D \le w_0 \\ \dfrac{2}{w_0 + w_S}\left(1 - \dfrac{D - w_0}{w_S}\right) & D > w_0 \end{cases} \qquad \text{(S7.5)}$$

and

$$f_S(D) = \begin{cases} \dfrac{1}{w_S} & w_0 \le D \le w_0 + w_S \\ 0 & otherwise \end{cases} \qquad \text{(S7.6)}$$

Thus for $w_0 \le D \le w_0 + w_s$, $f_A(D) < f_S(D)$. Letting $g(D)$ be any monotonically increasing function of D, it is straightforward to show that

$$\begin{aligned} \left\langle g(D) \right\rangle_S &= \int_0^{w_0+w_S} g(r)\, f_S(r)\, dr = \int_{w_0}^{w_0+w_S} g(r)\, f_S(r)\, dr \\ &= \int_{w_0}^{w_0+w_S} g(r)\, f_A(r)\, dr + \int_{w_0}^{w_0+w_S} g(r)\left[f_S(r) - f_A(r) \right] dr \end{aligned} \qquad \text{(S7.7)}$$

Similarly,

$$\left\langle g(D) \right\rangle_A = \int_0^{w_0} g(r)\, f_A(r)\, dr + \int_{w_0}^{w_0+w_S} g(r)\, f_A(r)\, dr \qquad \text{(S7.8)}$$

The left hand expressions of 1.3 and 1.4 are the expected or average values of the function $g(D)$, which may be considered as an error metric. The latter justifies the property that it increases with the estimation error distance D.

Since $\int_{w_0}^{w_0+w_S} f_S(r) - f_A(r)\, dr = \int_0^{w_0} f_A(r)\, dr$, and $g(\cdot)$ is monotonically increasing,

$$\int_{w_0}^{w_0+w_S} g(r)\left[f_S(r) - f_A(r) \right] dr > \int_0^{w_0} g(r)\, f_A(r)\, dr \qquad \text{(S7.9)}$$

Adding $\int_{w_0}^{w_0+w_S} f_A(r)\,dr$ to both sides,

$$\begin{aligned}\langle g(D)\rangle_A &= \int_0^{w_0+w_S} g(r)\, f_A(r)\,dr \\ &< \int_{w_0}^{w_0+w_S} g(r)\, f_S(r)\,dr = \int_0^{w_0+w_S} g(r)\, f_S(r)\,dr = \langle g(D)\rangle_S\end{aligned} \tag{S7.10}$$

Hence using any error metric which increases monotonically with error distance, the average value of that metric is greater measured with respect to all points within the triangular region, than with respect to all points along the boundary segment. Applying the same definitions and arguments as for $\langle D^2\rangle$, it can be seen that

$$\langle g(D)\rangle_{S_{Total}} > \langle g(D)\rangle_{A_{Total}} \tag{S7.11}$$

Therefore, at a convex arena's boundary, a uniform area estimate of position is better than a uniform boundary estimate of position for any monotonic error metric $g(\cdot)$. On its own, the knowledge of being in contact with a featureless boundary does not provide sufficient information to yield an improved distributed estimate of position compared with no knowledge of boundary contact.

It is worth noting that the theoretical results presented here may not apply if the arena is not uniformly sampled. For instance, if for some reason one point along the arena boundary is visited much more frequently than all other parts of the boundary, then it is theoretically possible for a navigating agent which has this knowledge to localize accurately when it is in contact with the boundary. In other words, if it is at the boundary, it is most likely to be in one particular location along that boundary. Effectively, this provides similar localizing information to having a feature along the boundary. Similar arguments apply for heterogeneous sampling within the arena, if the navigating agent has knowledge of that sampling distribution.
