# Supplementary material for: Maintaining a Cognitive Map in Darkness: The Need to Fuse Boundary Knowledge with Path Integration
Source: PLoS Comput Biol. 2012 Aug 16;8(8):e1002651. doi: 10.1371/journal.pcbi.1002651 (PMC3420935; doi:10.1371/journal.pcbi.1002651)
Supplement: Text S8 — Asymptotic place stability index in a circular arena. (PDF) [file pcbi.1002651.s021.pdf]

S8 - Asymptotic Place Stability Index in a Circular Arena

It has already been shown that at a circular boundary, the knowledge of being at the boundary is not sufficient to maintain a place stability index above 0.5. It is possible that the combination of iPI with boundary information effectively provides the radial position of the animal at all times, but little else. In this way, the distributed pose estimate due to iPI is modified by boundary memory and boundary contact and therefore is not fully modular. To investigate this possibility, we assume perfect radial position information, and find the expected place stability index assuming homogeneous coverage of a circular arena.

Based on the above assumption and from the geometry of a circle, the radial density function of actual traversals is directly proportional to the radius.

$$f(r) = \frac{2r}{r_{circ}{}^{2}} \tag{S8.1}$$

We can rewrite the expected squared distance at radial position r with respect to the null hypothesis as

$$\left\langle D_0{}^2 \mid r \right\rangle = r^2 + r_{circ}{}^2 / 2 \tag{S8.2}$$

Similarly, the expected squared distance at radial position r with respect to a uniform distribution of points along the circle of radius r is

$$\left\langle D_r{}^2 \mid r \right\rangle = 2r^2 \tag{S8.3}$$

The latter expression represents the situation where the navigating agent has precise information about the current radial position. It is then straightforward to show that

$$\left\langle I_P \right\rangle = \int_0^{r_{circ}} f(r) \frac{\left\langle D_0{}^2 \mid r \right\rangle}{\left\langle D_0{}^2 \mid r \right\rangle + \left\langle D_r{}^2 \mid r \right\rangle} dr = \cdots = \frac{3 + \ln 7}{9} \approx 0.5495 \tag{S8.4}$$

Note that this value is independent of the radius of the circular arena. This represents the upper limit of expected place stability if the fusion of iPI and boundary information could only provide perfect radial position. The fact that the average place stability index remained above this value for 48 minutes shows that more than radial position information was available. This was also evident on inspection of the particle cloud during most simulations, where the particle distribution did not extend to include a full circular annulus at any radial position (e.g., Video S1 & S2). Over longer periods of time, the place stability index will eventually reach and fall slightly below this value. The latter occurs because a perfect radial position estimate is impossible given noisy iPI and noisy wall contact information.

At the last step of 1,000 trials of 48 minutes in a 76cm circular arena, the distribution of I_p values was significantly above this limit ($t_{999} = 11.17$, $p<10^{-100}$).

The scenario presented here may be considered as being the ideal case under a number of different conditions. Firstly, it represents the upper bound of expected place stability index $\langle I_p \rangle$ after an extremely long period without vision (or other localizing cues other than boundary information). This is due to the fact that the positional uncertainty distribution tends to spread in a systematic way so that it approaches a circular annulus, concentric with the circular boundary (see Video S1 & S2 for an example of the start of this dynamic process). In essence, the simulated rat will have no information about its current location other than its radial position. When it is close to the arena center, the radial position alone is sufficient for accurate localization, leading to a high I_p approaching unity. Close to the boundary, this value has been shown to drop to 3/7 (Table S2). Assuming a uniform coverage of the arena, average place stability index $\langle I_p \rangle = (3 + \ln 7)/9 < 0.55$ (Eq S32). This represents an upper bound since Eq S32 was derived assuming that radial position is known precisely, which cannot be the case using erroneous iPI and realistic boundary information.

An alternative interpretation is as follows. Suppose that a navigation system is capable of full fusion of all available sensory information in a Bayes-optimal manner. Suppose further that it uses a modular PI and boundary system when vision is available, but does not switch to the optimal information fusion system until after the first boundary contact without vision, i.e., the first boundary contact following the start of iPI triggers the system. Since the first boundary contact uses the modular navigation system, the posterior position distribution is a uniform distribution along the entire boundary. Subsequently, optimal fusion of iPI and boundary information can only provide radial position. Assuming a uniform coverage of the arena, the upper bound of $\langle I_p \rangle$ is again as per Eq S32. This result highlights the fact that in at least some navigation tasks, it is important to combine information in a near optimal manner continually, rather than sporadically.
