# Supplementary material for: Maintaining a Cognitive Map in Darkness: The Need to Fuse Boundary Knowledge with Path Integration
Source: PLoS Comput Biol. 2012 Aug 16;8(8):e1002651. doi: 10.1371/journal.pcbi.1002651 (PMC3420935; doi:10.1371/journal.pcbi.1002651)
Supplement: Text S9 — Varying simulated place field locations. (PDF) [file pcbi.1002651.s022.pdf]

S9 – Varying Simulated Place Field Locations

Place fields centered at different radial positions in circular arenas were simulated using identical trajectories and particle cloud histories. All fields were averaged from the most stable 10% of trials based on the place stability index. Fields closer to the center of the arena showed higher spatial information content, raw and maximum spatial correlation [43], and spatial coherence (Fig S4 & S5, Table S3).

Note that using the polar mean for particle cloud distributions at or close to (0,0) causes overestimation of the 'radial' position since the mean radius reflects the width of the distribution itself. Consequently, the total spike count is lowered by this property of the polar mean for the simulated field radial position at 0cm.

These systematic variations in spatial properties were largely unrelated to directional information (Fig S4, S5). However, simulated fields near boundaries did appear to have more directional information content, particularly using the Cartesian mean.

The positional distribution near a circular boundary typically took on the curvature of the boundary (see Video S1 & S2). The Cartesian mean tended to underestimate the radial position of such a distribution. Consequently, the estimated position was less frequently near the arena boundary than using the polar mean (Fig S6I), leading to a reduced spike count. The reason for the preferred direction in the peripheral fields using the Cartesian mean is due to the fact that in order for the Cartesian mean to be close to the boundary, the cloud distribution had to be practically adjacent to the boundary. The latter was more likely when the animal was heading towards the boundary, since any subsequent step was likely to bring the animal too close to the center of the arena.

A potential disadvantage of using the polar mean as a measure of the center of a particle cloud is the overestimation of the radial position of cloud distributions near the arena center. This occurs because the radial mean of a distribution at the arena center is not zero, but is in fact the mean radial spread of the distribution. Hence it is relatively less common for a polar estimate to be close to the arena center, compared to a Cartesian estimate of particle cloud position. Consequently, the spike count was reduced for simulated place fields at the center of the circular arena using the polar mean compared to using the Cartesian mean (Table S3).

It is interesting to note that in representational space, the arena was not sampled homogeneously with respect to the traversed space, using either method, in either the circular or the square arena (Fig S6I & S6J). Nevertheless, both methods were successful in generating simulated fields with high spatial selectivity, in both arenas.
