# Supplementary material for: Maintaining a Cognitive Map in Darkness: The Need to Fuse Boundary Knowledge with Path Integration
Source: PLoS Comput Biol. 2012 Aug 16;8(8):e1002651. doi: 10.1371/journal.pcbi.1002651 (PMC3420935; doi:10.1371/journal.pcbi.1002651)
Supplement: Text S10 — Maintaining place stability in square versus circular arenas. (PDF) [file pcbi.1002651.s023.pdf]

S10 – Maintaining Place Stability in Square Versus Circular Arenas

Fig 3D & S6 show that the average place stability index is higher in square arenas than in circular arenas, under the same conditions. This is due at least in part from the use of wall contact information to re-orient the particle cloud with respect to the nearest wall. Assuming a particle cloud implementation as described in Methods, we consider the potential influence of different arena shapes when the simulated rat is in contact with the arena wall.

Initially, the cumulative positional and directional errors are small. In a square arena, the nearest wall in representation is likely to be the correct wall, which means that re-orientation effectively nullifies past cumulative heading errors. In other words, the majority of the particle cloud re-orients with respect to a particular wall, whose allocentric orientation is fixed. In a circular arena, the nearest point on the boundary cannot provide absolute orientation since a conjunctive shift in position and orientation could result in an equally valid relative wall contact vector as the correct pose, leaving that particle uncorrected. Over time, the particle cloud spreads and drifts, leading to positional uncertainty and low place stability.

By choosing the most stable 10% of trials, there was purposefully a selection bias. Three important effects of this bias are described. Firstly, choosing trials on the basis of low average I_p values did not selectively bias towards trials in which the model HD error distribution variance was particularly low. For example, at the last step of the 48-minute trials shown in Fig S6E-H, there was no significant increase in the variance of HD error in 100 random trials compared to the most stable 100 trials, in either the circular arena (Fig S6E, S6G; $f_{99,99} = 1.25$, $p = 0.13$) or in the square arena (Fig S6F, S6H; $f_{99,99} = 0.99$, $p = 0.52$).

Secondly, the highest place stability values were associated with trials where the particle filter estimates of heading were closer to the true heading than the corresponding HD model (Fig S6E, S6F). This phenomenon was particularly obvious in the square arena (Fig S6F). The clear differences between the particle filter heading error distribution (red) and model HD error distribution (grey) shows that the top 10% of trials were successful at reducing the error in estimated heading, and did not simply follow the simulated HD error distribution.

Thirdly, in random samples from the circular arenas, the particle filter estimate was approximately as erroneous as the model HD system (Fig S6G). The latter result shows that the above-chance average I_p values shown previously (Fig 3 & S6A) could not simply be attributed to a reduction in the particle filter estimate of heading. However, in random samples from the square arenas, the particle filter estimate had lower error than the model HD system (Fig S6H). This at least partly accounts for the higher average I_p values in square arenas compared to circular arenas of equivalent size.

These results are in agreement with existing literature on navigation models using particle filters, showing that near-optimal navigation involves a complex interaction between position and heading estimates (pose).

It is further evidence that position and heading representations should be considered concurrently rather than independently in the context of spatial navigation.

Finally, it is worth noting that detection of arena corners could not be modelled explicitly in this study without making further assumptions about the boundary contact process e.g. extent of contact sweep. For simplicity, we assumed that the rat detected the closest point along the boundary. Since all interior positions within a convex polygon are closer to points along boundary edges than vertices, the simulated rat in rectangular arenas never detected a vertex. Nonetheless, the broader corner geometry (convergence of two walls) tended to cause collapse of the particle cloud, when the agent randomly traversed between adjacent walls of a corner. The uncertainty distribution collapsed into (approximately) a line against one wall, and then collapsed further into (approximately) a point against the adjacent wall. In the absence of catastrophic failure (see above), this phenomenon produced a high level of place stability. In fact, corners represent very specific locations along a boundary and may be considered as point landmarks, even if not unique. A navigation agent able to explicitly recognized corners could, in principle, localize more accurately during contacts than our modelling suggests.
