# Supplementary material for: Maintaining a Cognitive Map in Darkness: The Need to Fuse Boundary Knowledge with Path Integration
Source: PLoS Comput Biol. 2012 Aug 16;8(8):e1002651. doi: 10.1371/journal.pcbi.1002651 (PMC3420935; doi:10.1371/journal.pcbi.1002651)
Supplement: Text S11 — Path integration with vision but no boundary map. (PDF) [file pcbi.1002651.s024.pdf]

S11 – Path Integration with Vision but no Boundary Map

We investigated the computational plausibility of using PI with vision but no boundary map to maintain a stable place field. Here, the HD system was assumed to have the same magnitude of random error per step, but that the HD drift was zeroed following each step. Otherwise, all simulations were as previously described for PI-only navigation. We found that it was impossible to maintain place stability with aPI alone. These results imply the need to use allothetic cues such as arena boundaries even if aPI is possible. Simulated place firing is shown in Fig S7, and firing statistics shown in Table S4.

It is worth noting that the spatial information content remained relatively stable despite a clear drop in the pixel-by-pixel correlation between the first and subsequent fields, as well as a decrease in the total number of spikes (discussed in main text). The low spike count relative to the number of spatial pixels makes the estimation of pixel-wise firing probabilities noisy, which tends to cause an overestimation of the spatial information content of the firing field. This problem may be reduced by increasing the total spike count by simulating spikes between step end points, but requires further assumptions about the nature of the spike process itself, as well as interpolation of the state of the navigation system between steps.
