# Supplementary material for: Maintaining a Cognitive Map in Darkness: The Need to Fuse Boundary Knowledge with Path Integration
Source: PLoS Comput Biol. 2012 Aug 16;8(8):e1002651. doi: 10.1371/journal.pcbi.1002651 (PMC3420935; doi:10.1371/journal.pcbi.1002651)
Supplement: Text S12 — Grid fields without vision following arena compression and expansion. (PDF) [file pcbi.1002651.s025.pdf]

S12 – Grid Fields Without Vision Following Arena Compression and Expansion

Based on the experiments of [51], we simulated grid fields where testing occurred in four different arenas, only one of which was identical to the training arena (Fig 6 & 7). A key finding of [51] was that the grid spacing rescaled partially, in the same direction as the arena deformation.

Using two variants of the particle filter model described earlier, we simulated grid fields without vision. It was found that stable grid-like firing patterns were seen in most combinations of training arena, test arena and particle filter variant. However, there were clear differences with respect to the results of [51] where visual information was available to the animal.

In simulations where the test arena was expanded relative to the training arena, grid field splitting was observed, similar to place field splitting described earlier. This was not unexpected for similar arguments as presented for place field splitting. This model predicts that if only idiothetic and featureless boundary cues are present, grid fields should split in expanded test arenas, assuming grid cells represent an array of relative locations and operate by optimally combining iPI and a fixed stored arena boundary map.

In simulations where the test arena was compressed relative to the training arena, the pattern of simulated grid fields depended on the particle filter algorithm used. Using standard stochastic universal resampling, arena compression had the effect of generating two partial fields, which depended on the last boundary contact. Using resampling where only heading was updated, partial grid compression was found.

To examine these phenomena in more detail, the relationship between the estimated position and true position was examined along the axis of arena expansion or contraction between two reciprocal transformations (Fig S8).

In all cases, there was a strong correlation between the estimated X position and true X position when the test arena was consistent with the training arena (black points). This result is consistent with the finding of high place stability in square arenas (special case of rectangular arenas) reported earlier, in which place fields of high spatial information content were generated in simulation.

When the test arena was compressed in the X direction (Fig S8A & S8B), the relationship between estimated X position and true X position depended on the particle resampling method used. Using standard stochastic universal resampling, the relationship was split into two distinct clusters, shifted approximately by the arena compression (30cm) in the estimated X position. This phenomenon may be explained as follows. The arena in memory was 100cm in length along the X axis, whereas the true arena was only 70cm. The result was that when the simulated rat reached a true boundary (X_{true}=±35cm), in representational space, it had not reached

either boundary in memory ($X_{boundary}$=±50cm). However, only particles which by chance happened to be close to $X_{boundary}$=±50cm were assigned high importance (relatively more consistent with boundary contact information). This led to large jumps in the estimated position each time the simulated rat reached a true boundary. Once this occurred, the estimated X position was again correlated with the true X position (due to iPI), shifted by approximately ±15cm, until the next encounter with the opposite boundary.

The simulated grid fields were effectively an overlay of two portions of the original grid, one ranging from approximately -50cm < X < +20cm, the other ranging from approximately -20cm < X < +50cm. By chance, this was still grid-like in some instances.

Using stochastic universal resampling but where only particle heading was modified, the split relationship between estimated X position and true X position was not evident. This can be explained as follows. Since the positions of particles were not changed due to boundary contact, there were no large jumps in position estimate, but the particle cloud estimate was noisy. However, since the arena extent in memory was larger than the true arena extent (in the X direction), particles which exceeded X=±35cm were not culled. Over time, this meant an outward drift in estimated X position with respect to true X position, partially constrained by iPI, and eventually curbed by $X_{boundary}$=±50cm in memory if they drifted too far. In effect, the simulated navigation system had a noisy estimate of X position ranging larger than X=±35cm but smaller than X=±50cm.

The simulated grid fields were effectively a sample of the original grid somewhere between 70cm and 100cm in width, expressed in an arena 70cm wide. Consequently, partial field compression was observed when spikes were shown with respect to true X position.

When the test arena was expanded in the X direction (Fig S8C & S8D), the relationship between estimated X position and true X position also depended on the particle resampling method used, but to a lesser extent than during arena compression. In both cases, the relationship between estimated X position and true x position was split. This was a different phenomenon to that seen in arena compression. Here, particles have reached the boundary in memory, but true boundary contact had not occurred. Consequently, particles outside of the memorized arena extent, i.e, |X|>35cm, were culled. In essence there was no jump in estimated X position. Rather, the estimated X position remained almost static if the simulated rat persisted beyond the extent of the boundary in memory.
The simulated grid fields were effectively an overlay of two full original grids (70cm wide), one at true location range -50cm < X < +20cm, and one at true location range -20cm < X < +50cm. Due to the spatial phase shift of approximately 30cm between the two grids, a split grid field was produced (in a manner similar to simulated split place fields).

The main difference due to the particle resampling method used seemed to be the probability of rotational errors occurring. Rotational errors may be thought of as ‘correct’ pose relative to an ‘incorrect’ wall. Suppose a simulated rat made contact with the East wall (X_{true}=35cm), but the particles were still far from the East wall in memory ($X_{boundary}$=50cm). If the simulated rat happened to be close to the North or South wall, then the distance of particles to the North or South wall in memory may be closer than the distance to the East wall. Consequently, the posterior probability (particle weights) may be higher for those particles which were otherwise poor estimates based on heading, but which happened to be closer to the ‘wrong’ wall. In this way, the position estimate jumped to a different wall, causing a rotational error. When particle position was not updated, there was a reduced tendency for the position estimate to jump. Consequently, the simulated fields showed less spatial regularity or specificity. The latter phenomenon was particularly evident when there was a large discrepancy along both spatial dimensions between the training and test arena (e.g., Fig 5A & 5C lower right panels).
