# Supplementary material for: Maintaining a Cognitive Map in Darkness: The Need to Fuse Boundary Knowledge with Path Integration
Source: PLoS Comput Biol. 2012 Aug 16;8(8):e1002651. doi: 10.1371/journal.pcbi.1002651 (PMC3420935; doi:10.1371/journal.pcbi.1002651)
Supplement: Text S13 — Animated example of simulated localization without vision. (PDF) [file pcbi.1002651.s026.pdf]

S13 – Animated Example of Simulated Localization Without Vision

Two videos showing the first (Video S1) and last (Video S2) 8 minutes of an example of simulated localization using a particle filter in darkness over 48 minutes, in a 76cm diameter circular arena. See Methods and S1-S3 for details of the HD error model and particle filter implementation. In each video, the true position of the simulated rat (red asterisk) and the particle cloud (blue dots) are superimposed on the trajectory (grey line). The particle cloud represents the navigation system's distributed estimate of current position.

Note that the first 40 frames of Video S1 were purposefully slowed to clearly show the increase in positional uncertainty (spread of particle cloud) during idiothetic path integration (iPI), and the decrease in uncertainty possible when boundary contact is made. The corresponding place stability index profile is shown in Fig S9.
