# Supplementary material for: Maintaining a Cognitive Map in Darkness: The Need to Fuse Boundary Knowledge with Path Integration
Source: PLoS Comput Biol. 2012 Aug 16;8(8):e1002651. doi: 10.1371/journal.pcbi.1002651 (PMC3420935; doi:10.1371/journal.pcbi.1002651)
Supplement: Text S14 — Utility of modular navigation models. (PDF) [file pcbi.1002651.s027.pdf]

S14 - Utility of Modular Navigation Models

Although the model of [13] was based on insect data, it is a proof-of-concept that a modular navigation system can function and explain real navigation data. It is straightforward to extend the model to practical navigation problems where, for instance, noise is significant. Take the example of optimal estimation of point landmark positions, which some authors consider as a “mapping” problem. Due to the central limit theorem, the positional estimation error converges to a bivariate Gaussian distribution [8,9]. Hence Bayes-optimal update of landmark positions using noisy PI here is simply a problem of weighting repeated PI position estimates with the inverse covariance matrix of the uncertainty distribution, i.e.,

$$\boldsymbol{\mu}_{Opt}\boldsymbol{\Sigma}_{Opt}^{-1} = \boldsymbol{\mu}_1\boldsymbol{\Sigma}_1^{-1} + \cdots + \boldsymbol{\mu}_t\boldsymbol{\Sigma}_t^{-1} \quad \text{(S14.1)}$$

where $\boldsymbol{\mu}_k$ is the PI estimate of landmark position due to the *k*th visit, and where the inverse of the optimal covariance matrix is given by the sum of the inverse covariances of the PI estimates from all past visits, i.e.,

$$\boldsymbol{\Sigma}_{Opt}^{-1} = \boldsymbol{\Sigma}_1^{-1} + \cdots + \boldsymbol{\Sigma}_t^{-1} \quad \text{(S14.2)}$$

Under some circumstances, the above results can be simplified further. It has been shown that in the presence of a compass cue (such as the E-vector for some insects), PI errors are expected to grow linearly with the number of steps taken, n, and whose covariance matrix eigenvectors have the same orientations relative to the main axis of locomotion [8,9]. This result is analytically exact for all repetitions of navigation journeys which follow the same path, and a close approximation for small to moderate deviations. Near-optimal estimation of landmark position simplifies to one of weighting repeated PI estimates by the inverse of the number of steps taken (since the inverse covariance matrices are now all proportional to the inverse of the step number), i.e.,

$$\boldsymbol{\mu}_{Opt}\left(\frac{1}{n_1} + \cdots + \frac{1}{n_t}\right) \approx \frac{\boldsymbol{\mu}_1}{n_1} + \cdots + \frac{\boldsymbol{\mu}_t}{n_t} \quad \text{(S14.3)}$$

Therefore, near-optimal update of landmark positions can be achieved iteratively without a map (only a step counter or stride integrator, which may or may not be separate to the PI module). Updating the optimal position estimate after visit *t* is achieved by a linear combination of the previous optimal estimate, $\boldsymbol{\mu}_{opt}(t-1)$ and the PI estimate from visit *t*, $\boldsymbol{\mu}_t$, so that

$$\boldsymbol{\mu}_{opt}(t) \approx \boldsymbol{\mu}_{opt}(t-1)\left(\frac{\frac{1}{n_1} + \cdots + \frac{1}{n_{t-1}}}{\frac{1}{n_1} + \cdots + \frac{1}{n_t}}\right) + \boldsymbol{\mu}_t\left(\frac{\frac{1}{n_t}}{\frac{1}{n_1} + \cdots + \frac{1}{n_t}}\right) \quad \text{(S14.4)}$$

Hence a direct extension of the modular model of [13] makes it a Bayes-optimal (or at least near-optimal) mapping system in the presence of noise, yet it can maintain full modularity during subsequent navigation tasks. Together with the fact that the navigation modules contain as much information as a map (see Introduction), it is not clear that a cognitive map model necessarily confers any significant advantage for spatial navigation, in the open field where compass information is available. Compass information is available, for instance, in rodents with vision as indicated by their stable HD system (stable HD cell tuning functions).

Modularity *per se* may be advantageous. Firstly, both insect and mammalian brains are anatomically modular, with certain regions serving distinct functions. Even the neuronal properties on which many of the arguments of our work are based are only found in specific regions of the rodent brain. Secondly, it is not clear whether a fully interconnected (and interdependent) network of neurons can evolve effectively if the function of each subregion depended on all other subregions. A similar principle applies to complex computer systems, where changes or modifications in function have to be possible without excessive side effects. Thirdly, a modular model which suffices to explain the relevant data can often be more readily understood, tested and modified.

For these reasons, it is prudent to consider to what extent neural navigation systems may be considered as functionally modular. It is specifically the combination of using boundary landmarks without a compass (and hence using iPI) which makes the navigation problem particular challenging as described in this work. We are not aware of any previous work which has quantitatively shown just how difficult this problem is for a modular navigation system, nor whether the combination of iPI and boundary information can make a sufficient difference to explain stable place and grid fields without vision.
